# Construction of Reference Chromosome-Scale Pseudomolecules for Potato: Integrating the Potato Genome with Genetic and Physical Maps

**DOI:** 10.1534/g3.113.007153

**Published:** 2013-11-01

**Authors:** Sanjeev Kumar Sharma, Daniel Bolser, Jan de Boer, Mads Sønderkær, Walter Amoros, Martin Federico Carboni, Juan Martín D’Ambrosio, German de la Cruz, Alex Di Genova, David S. Douches, Maria Eguiluz, Xiao Guo, Frank Guzman, Christine A. Hackett, John P. Hamilton, Guangcun Li, Ying Li, Roberto Lozano, Alejandro Maass, David Marshall, Diana Martinez, Karen McLean, Nilo Mejía, Linda Milne, Susan Munive, Istvan Nagy, Olga Ponce, Manuel Ramirez, Reinhard Simon, Susan J. Thomson, Yerisf Torres, Robbie Waugh, Zhonghua Zhang, Sanwen Huang, Richard G. F. Visser, Christian W. B. Bachem, Boris Sagredo, Sergio E. Feingold, Gisella Orjeda, Richard E. Veilleux, Merideth Bonierbale, Jeanne M. E. Jacobs, Dan Milbourne, David Michael Alan Martin, Glenn J. Bryan

**Affiliations:** * Cell and Molecular Sciences, The James Hutton Institute, Dundee DD2 5DA, United Kingdom; † Division of Biological Chemistry and Drug Discovery, College of Life Sciences, University of Dundee, Dundee DD1 5EH, United Kingdom; ‡ Laboratory of Plant Breeding, Department of Plant Sciences, Wageningen-UR, Wageningen, The Netherlands; § Department of Biotechnology, Chemistry and Environmental Engineering, 9000 Aalborg University, Aalborg, Denmark; ** International Potato Center (CIP), Lima 12, Peru; †† Laboratorio de Agrobiotecnología, Instituto Nacional de Tecnología Agropecuaria (INTA) cc276 (7620) Balcarce, Argentina; ‡‡ Laboratorio de Genética y Biotecnología Vegetal, Universidad Nacional San Cristobal de Huamanga, Ayacucho, Perú; §§ Mathomics, Centro de Regulación Genómica & Centro de Modelamiento Matemático, Universidad de Chile, Santiago, Chile; *** Department of Crop and Soil Sciences, Michigan State University, East Lansing, Michigan; ††† Genomics Research Unit, Facultad de Ciencias, Universidad Peruana Cayetano Heredia, Lima 31, Peru; ‡‡‡ Institute of Vegetables, Shandong Academy of Agricultural Sciences, Jinan 250100, China; §§§ Biomathematics and Statistics Scotland, Dundee DD2 5DA, United Kingdom; **** Department of Plant Biology, Michigan State University, East Lansing, Michigan; †††† Institute of Vegetables and Flowers, Chinese Academy of Agricultural Sciences, Beijing 100081, China; ‡‡‡‡ Information and Computational Sciences, The James Hutton Institute, Dundee DD2 5DA, United Kingdom; §§§§ INIA-La Platina, Santiago, Chile; ***** Crops Environment and Land Use Programme, Teagasc, Carlow, Ireland; ††††† The New Zealand Institute for Plant & Food Research Ltd., Christchurch 8120, New Zealand; ‡‡‡‡‡ INIA-Rayentué, Rengo, Chile; §§§§§ Department of Horticulture, Virginia Polytechnic Institute and State University, Blacksburg, Virginia 24061

**Keywords:** Solanaceae, genome anchoring, scaffold orientation, sequence-tagged sites, pseudomolecules, potato, genetic map, physical map

## Abstract

The genome of potato, a major global food crop, was recently sequenced. The work presented here details the integration of the potato reference genome (DM) with a new sequence-tagged site marker−based linkage map and other physical and genetic maps of potato and the closely related species tomato. Primary anchoring of the DM genome assembly was accomplished by the use of a diploid segregating population, which was genotyped with several types of molecular genetic markers to construct a new ~936 cM linkage map comprising 2469 marker loci. *In silico* anchoring approaches used genetic and physical maps from the diploid potato genotype RH89-039-16 (RH) and tomato. This combined approach has allowed 951 superscaffolds to be ordered into pseudomolecules corresponding to the 12 potato chromosomes. These pseudomolecules represent 674 Mb (~93%) of the 723 Mb genome assembly and 37,482 (~96%) of the 39,031 predicted genes. The superscaffold order and orientation within the pseudomolecules are closely collinear with independently constructed high density linkage maps. Comparisons between marker distribution and physical location reveal regions of greater and lesser recombination, as well as regions exhibiting significant segregation distortion. The work presented here has led to a greatly improved ordering of the potato reference genome superscaffolds into chromosomal “pseudomolecules”.

Genome sequencing of crop plants has become increasingly routine, primarily due to the reduction in cost and increase in throughput brought about by continuing advances in sequencing technologies. First reports on the whole-genome sequences of plants, such as *Arabidopsis thaliana* (The Arabidopsis Genome Initiative 2000) and rice (International Rice Genome Sequencing Project 2005), were mainly accomplished with the use of clone-based (*e.g.*, “BAC by BAC”) strategies. In this approach, a library of bacterial artificial chromosome (BAC) clones is mapped onto chromosomes by the use of molecular markers, the aim being to generate a clone-based physical map with a “minimum tiling path.” This assures good genome coverage while minimizing the sequencing effort. More recently, plant genome sequencing has been based on whole-genome shotgun approaches involving conventional Sanger sequencing, next-generation sequence technologies, or a combination of both (Hamilton and Buell 2012). The whole-genome shotgun approach does not require a physical map, and there is no preassumption of the position of the resulting sequence assemblies. Several research groups have developed “scaffolding” algorithms to assemble these typically short sequence contigs into larger constructs (Miller *et al.* 2010). However, because of the genome size and complexity of most crop plants, scaffolds typically remain unoriented and without chromosomal coordinates, despite being well annotated for gene content. A reference genome sequence requires that the products of the assembly process (contigs and scaffolds) be globally ordered and oriented to generate chromosomal pseudomolecules (PMs). In the absence of a clone-based physical map or genetic map of the reference sequenced genotype, this task is a significant and challenging one. One widely adopted approach has been to link the sequence assembly to a genetic map using the presence of mapped sequence-tagged site (STS) genetic markers (Green and Green 1991) in the genome sequence. For example, a set of 409 molecular markers was used to order 69% of the assembled 487 Mb grapevine genome along the 19 grape linkage groups (The French-Italian Public Consortium for Grapevine Genome Characterization 2007). The link between the genome sequence and its genetic maps is critical in moving between trait loci and candidate genes underlying such loci. Successful genetic anchoring of a plant genome sequence assembly with the use of maps developed in the reference-sequenced genotype depends on marker density and distribution, as well as map accuracy and resolution. Other approaches can also be implemented to augment the anchoring process, including comparative analysis with physical and genetic maps of closely related species.

The Potato Genome Sequencing Consortium (Potato Genome Sequencing Consortium 2011) has published the genome of the doubled monoploid *Solanum tuberosum* group Phureja DM1-3 516 R44 (hereafter referred to as DM). At the time the genome sequencing was initiated, DM did not have a physical map, nor was there any pre-existing genetic map for this genotype. Therefore, a genome-anchoring strategy was developed that included the generation of a segregating bi-parental mapping population involving DM as a parent, and generation of a dense STS-based genetic map. Other genetic mapping resources, such as the ultra-high density (UHD) map of diploid potato genotype RH89-039-16 (RH) (van Os *et al.* 2006), and the tomato-EXPEN 2000 genetic reference map (Fulton *et al.* 2002) were also used.

We describe for the first time in detail the generation of an integrated *de novo* genetic/physical map of potato and significant refinements to the previously published assembly. Our combined map orders the genome sequence into 12 chromosomal PMs corresponding to each of the 12 potato chromosomes and is linked to previously existing potato and Solanaceae mapping resources. The work represents the assimilation of various data types that required complex interpretation for correct ordering and orientation of superscaffolds. This process involved considerable manual curation, driven largely by a novel approach for visualization of mate-pair sequences from large genomic clones (BAC and fosmid) and long insert 454 reads (20 kb and 8 kb). This allowed us to assign robust orientations to many superscaffolds and also enabled the inclusion of many superscaffolds that remained unanchored when the reference genome sequence was published (Potato Genome Sequencing Consortium 2011). This resource will facilitate exploitation of the potato genome sequence for genetic analysis and crop improvement, and our approach can serve as a guide for others wishing to engage in genome sequencing of genotypes which lack physical or genetic maps.

## Materials and Methods

### Genetic cross/population construction

A segregating diploid potato population (BC_1_) derived from the reference sequence clone DM 1-3 516 R44 (DM) was developed. The homozygous DM clone (2*n* = 2*x* = 24) was generated by chromosome doubling of a monoploid (2*n* = 1*x* = 12) derived from a heterozygous accession of *S. tuberosum* Group Phureja (Paz and Veilleux 1999). A heterozygous diploid clonal accession (CIP 703825, referred to as D) belonging to the *Solanum tuberosum* diploid Andigenum Group Goniocalyx cultivar group (Spooner *et al.* 2007; Ovchinnikova *et al.* 2011) was crossed to DM. The direction of the cross (DM × D) was chosen because DM is male sterile. One of the resulting F_1_ hybrids (DM/D, CIP 305156.17) was used as the stylar parent in a backcross with D as pollen parent. The mapping population comprising 180 backcross progeny clones (hereafter referred to as DMDD) was raised in the greenhouse for DNA extraction and pathogen testing and is also maintained pathogen-free *in vitro* (https://research.cip.cgiar.org/confluence/display/dm/Home) at the International Potato Center, Peru.

### Plant material and genomic DNA extraction

Genomic DNA from 180 progeny clones of the mapping population and the pedigree parents was isolated by the use of standard protocols (Herrera and Ghislain 2000). DNA concentration was estimated with a TBS-380 Fluorometer (Turner BioSystems) with PicoGreen reagent using salmon sperm DNA at 500 ng/mL as a reference. All DNA samples were normalized to a final concentration of 250 ng/µL and distributed among members of the Potato Genome Sequence Consortium (PGSC) mapping group to perform multilocation genotyping by using diversity arrays technology (DArT), simple sequence repeat (SSR), single-nucleotide polymorphism (SNP), and amplified fragment-length polymorphism (AFLP) markers.

### Marker identification, development, and analysis

#### SSR markers:

SSR markers were designed from an early draft of the assembled potato genome superscaffolds (DM assembly version 1). Markers were selected from a masked copy of the genome to avoid placement in repetitive DNA. In addition to these SSR markers (labeled PM), previously reported sets of SSRs from Stwax (potato *waxy* gene; Veilleux *et al.* 1995), STM (Milbourne *et al.* 1998), STI (Feingold *et al.* 2005), st_ (Tang *et al.* 2008a), and STG (Ghislain *et al.* 2009) were also used in linkage mapping. In total, 356 SSRs (Supporting Information, Table S1A) were tested for polymorphism. In brief, 5−25 ng of template DNA was added to polymerase chain reaction (PCR) mix containing 1.5−2.5 mM MgCl_2_, 0.16−0.25 mM dNTP, 0.25−1.0 U Taq polymerase, with the following primer combinations; for acrylamide gel analysis, 0.2−0.25 µM forward primer, 0.2−0.25 µM reverse primer, plus 0.2 mM cresol red and 6% sucrose; for ABI3130lx Genetic Analyzer (Applied Biosystems), 0.2−0.25 µM reverse primer, 0.15−0.25 µM forward primer, 0.05−0.25 µM labeled (FAM (5-FAM (6-FAM) 5(6)-carboxyfluorescein), HEX (6-carboxy-1,4-dichloro-2′,4′, 5′, 7′-tetrachlorofluorescein), NED, or PET) forward primer; for 4300 LI-COR DNA Analyzer (LI-COR Biosciences), 0.2 µM or 22 pM forward primer, 0.2 µM or 15 pM reverse primers, 25 pM 700 or 800 IRDye labeled M13 forward primer. PCRs were conducted under optimized conditions: in brief, 4 min denature at 94°, 35 cycles of 30 sec at 94°, 30 sec at T_a_ (annealing temperature determined experimentally for each SSR primer combination), 30 sec at 72°, 1 cycle of 4 min at 72°; or 3 min denature at 94°, 36 cycles of 15 sec at 94°, 30 sec at 58−52° with touchdown of −0.5° for first 12 cycles, 30 sec at 72°, 1 cycle of 5 min at 72°; or 4 min denature at 94°, 30−33 cycles of 1 min at 94°, 1 min at T_a_, 1 min at 72°, 1 cycle of 4 min at 72°. SSRs were resolved either by denaturing acrylamide gel electrophoresis and silver staining according to Creste *et al.* (2001), capillary electrophoresis following standard procedures for the ABI3130lx Genetic Analyzer using Genscan 400 ROX (6-carboxy-X-rhodamine) or Genscan 500 LIZ size ladder, or by electrophoresis on the 4300 LI-COR DNA Analyzer system (LI-COR Biosciences) using the LI-COR IRDye 50–350 bp size standard. Polymorphic markers were scored directly from silver stained gels; using GeneMarker 1.4 (SoftGenetics, State College, PA; www.softgenetics.com), GeneMapper 4.0 (Applied Biosystems) or Genographer (www.genographer.com) for ABI3130 lx; or the SAGA Generation 2 software (LI-COR, USA), and Cross Checker v.2.9.1 (Buntjer 1999) for LI-COR. SSRs were scored, where possible, as codominant markers, and if this was not possible, as dominant markers.

#### SNP markers:

A custom filtering pipeline was developed to select 1920 SNPs from a set of 69,011 high-confidence SolCAP SNPs (Hamilton *et al.* 2011) that were incorporated into five 384-plex (5 × 384) Illumina GoldenGate oligonucleotide pool assays (OPAs; Fan *et al.* 2003), hereafter referred to as POPA (potato OPAs). Hamilton *et al.* (2011) identified these SNPs by comparing RNA-Seq and EST sequences from six potato cultivars (Atlantic, Premier, Snowden, Bintje, Kennebec, and Shepody) to the draft DM potato reference genome. Our filtering pipeline involved finding nonrepetitive positions on the DM assembly, avoiding overlapping SNPs that may have interfered with the Illumina SNP genotyping assay, and striving to cover the genome as fully as possible. In addition, a POPA containing SNPs derived from pre-existing potato ESTs in the public databases was also designed and used. Table S1B shows details of 2304 SNPs, derived from pre-existing potato ESTs (POPA1) and SolCAP markers (POPA2-6) used in the study. Genotyping was performed using an Illumina BeadXpress platform following the recommendations of the manufacturer (GoldenGate Genotyping Assay, Illumina VeraCode Manual, VC-901-1001). All reagents, unless stated otherwise in the standard protocol, were provided by Illumina. The data files were processed and genotypes called using Genome Studio software.

#### AFLP markers:

AFLP analysis was performed according to the procedures described by Vos *et al.* (1995) using the restriction enzyme combination *Eco*RI and *Mse*I. AFLP fragments were separated on a LI-COR 4300 DNA Sequencer (LI-COR Biosciences) using 4.5% polyacrylamide denaturing gels (acrylamide:bisacrylamide, 19:1) as described in the user manual. The LI-COR size standard ladder was loaded into each lane to facilitate the semiautomatic analysis of the gel and the sizing of the fragments. The names of the markers indicate the enzymes used, the selective nucleotides, and the size of the fragment; for instance, EACTMAAC_205.0 is an AFLP marker derived from a primer combination with the enzymes *Eco*RI and *Mse*I, selective nucleotides ACT and AAC, and a mobility that corresponds to a fragment with an estimated size of 205 bp. Polymorphic bands were manually scored following the intensity degree and the parent backcross pattern. The details of the enzyme combinations, selective nucleotides, and adapter sequences are provided in Table S1C.

#### DArT markers:

Representations from 180 DMDD progeny clones and the pedigree parents (DM, DM/D, D) were obtained by subjecting DNA from each clone to double restriction enzyme digestion *(Pst*I/*Taq*I) and ligation to *Pst*I adaptors for reducing genome complexity followed by PCR amplification for preparation of targets (Wenzl *et al.* 2004). Cy3-labeled representations (targets), mixed in an ExpressHyb buffer containing cy5-labeled polylinker fragment of the plasmid used for library preparation (as a reference), were denatured and hybridized to a high-resolution potato genotyping array containing 7680 DArT probes (Sliwka *et al.* 2012). After overnight hybridization at 62°, arrays were washed and scanned with 20 μm resolution at 543 nm (cy3) and 488 nM (FAM) on a LS300 confocal laser scanner (Tecan, Grödig, Austria) to detect fluorescent signals emitted from the hybridized fragments. The data from the scanned images were extracted and analyzed using the DArTsoft 7.4 software (Diversity Arrays Technology P/L, Canberra, Australia). The logarithm of the ratio between the two background-subtracted averages of feature pixels in the cy3 and cy5 channels (log2[cy3/cy5]) was used as a measure of the difference in abundance of the corresponding DNA fragment in the two representations hybridized to the array. The log2[cy3/FAM] and log2[cy5/FAM] values, which are approximate measures of the amount of hybridization signal per amount of DNA spotted on the array, were used for quality-control purposes. The unique signal pattern obtained by hybridizing each sample pair (individual clone and reference) to the genotyping array was recorded as “0” or “1.” All DArTs were sequenced and are available from Spud DB site (http://potato.plantbiology.msu.edu/); the detailed methodology is published on the Diversity Arrays Technology website (http://www.diversityarrays.com).

### Linkage map construction

The SSR, SNP, AFLP, and DArT genotyping data for 180 DMDD progeny clones were combined and screened for polymorphic markers. JoinMap4 (Van Ooijen 2006) was used both to assign markers to linkage groups and to order markers within linkage groups. The backcross parents and offspring were coded according to the cross-pollinated (CP) population type (outbreeder full-sib family after two independent meioses). A female-male combined DMDD map was generated that included markers informative in one or both parents. Linkage groups were formed using the Independence LOD parameter under “population grouping” with a range from 2 to 15. Before grouping and ordering markers within linkage groups, loci or progeny clones with ≥20% missing values were removed along with all identically segregating loci. The regression mapping algorithm with modified settings (recombination frequency threshold < 0.49, LOD threshold > 0.01) was used to order loci within each linkage group. All linkage groups were subjected to three rounds of mapping. Recombination frequencies were converted into map distances using the “Kosambi” mapping function.

### Locating STS markers on the DM assembly

STS markers were aligned to the reference genome assembly using SSAHA2 (Ning *et al.* 2001) or BLAST. The total set of alignments was processed as follows. First, alignments caused by short repetitive sequences were removed using a custom depth/coverage filter. In detail, any alignment covering a region of the query or target sequence that overlapped with five or more other competing alignments in that region was removed if this depth threshold was exceeded greater than 20% or more of the alignment length. In this way alignments spanning short repeats were not penalized, but alignments largely composed of likely repeats were removed. Second, short alignments were grouped by sequence into “hits” that allowed for indels. Third, where applicable, the relative distance and orientation of the forward and reverse reads for the marker was taken into consideration. Pairs of forward and reverse reads with an incorrect orientation or implausible separation were removed. Finally, only markers with a unique, high-scoring alignment position on the genome assembly were selected as anchor points in the physical map. The final positions of all the STS markers (SSRs, SNPs, and DArTs) are provided in Table S2.

### Integration of additional sequence-based and physical resources

DM BAC- and Fosmid-end sequences, RH BAC-end sequences, and tomato BAC- and Fosmid-end sequences were aligned to the DM superscaffolds using SSAHA2 (Ning *et al.* 2001). The resulting alignments were filtered as described previously. Roche 454 Paired-end (PE) reads from 14- and 20-kb insert-size libraries from DM, representing 0.7 and 1.0 Gb of raw data, respectively, were aligned to the superscaffold sequences using Newbler (Margulies *et al.* 2006) with all the default settings. Unsequenced BAC clones from the RH physical map (de Boer *et al.* 2012) were positioned on the superscaffolds using BLAST alignment of their whole-genome profiling (WGP) sequence tags. For each BAC, the alignment hits of the individual 25 nt tags were processed to retain only unique hits. The aligned BAC clones that carried AFLP markers provided the link between the DM superscaffolds and the RH UHD genetic map (van Os *et al.* 2006). In addition, sequenced RH BAC clones and RH BAC-end sequences were used for anchoring and scaffolding of the DM sequences. Finally, sequences from the available tomato PMs (v2.40, The Tomato Genome Sequencing Consortium 2012) were aligned using ATAC (Istrail *et al.* 2004).

### Manual scaffolding using the “link-peak” strategy

All paired-end and mate-pair (PEMP) sequence data that could be reliably mapped to the DM superscaffolds were combined to compute a composite directional link-score across each superscaffold. In detail, the link-score combined PEMPs that had unique, high-scoring alignments for both ends of each mate pair sequence, but with the two end sequences aligning to different non-adjoining superscaffolds. A reciprocally high link-score between the ends of a pair of superscaffolds indicated a probable scaffolding link between them. The composite directional link-score is calculated in a sliding window along the length of a superscaffold (the source) as follows:

1. All mate pairs with one end aligning in that window and the other corresponding mate pair end reliably mapping to another superscaffold (the target) are selected. These are designated as unsatisfied mate pairs.
2. These mate pairs are grouped according to the target superscaffold.
3. For each target superscaffold group, a score is calculated by summing the value for each mate pair in that group (see below for details of how the value is determined).
4. The link-peak score is the greatest score of all the target groups.

Different link-score values were empirically assigned to the different PEMP sequence libraries, with greater scores assigned to DM based libraries over RH and tomato-based libraries and greater values given to longer sequences that have more accurate alignments. In addition to accumulating link-evidence from consistent unsatisfied PEMPs, a noise-score was calculated for unsatisfied PEMPs that suggested links to multiple different target superscaffolds. The noise score allowed spurious, high-scoring links caused by repeats to be identified. In this way the evidence for links between pairs of superscaffolds could be conveniently described as a continuous value in wiggle format (https://www.genome.ucsc.edu/goldenPath/help/wiggle.html), which allows for visualization as tracks in GBrowse, alongside genetic and physical evidence from other sources.

### Visualization of integrated genetic and physical map

The integrated genetic and physical maps of the DM genome were visualized with the software ‘DMAP’ (D. M. A. Martin, unpublished data). The figures produced by the DMAP software take as input the accessioned golden path (AGP) file describing the PM architecture, a GFF file describing the sequence positions of the markers on the superscaffolds, and the JoinMap output file from linkage mapping for each linkage group. As there are many more markers than those that can be coherently visualized on a printed figure, DMAP employs a selection and layout algorithm where only a user determined maximum number of labels are displayed.

DM chromosome idiogram figures were reproduced from the potato reference genome publication (Potato Genome Sequencing Consortium 2011) and were aligned by orienting the short arms toward the start of the PM sequence, except for chromosomes 5 and 11, where the PM sequence begins in the long arm (Tang *et al.* 2009; Potato Genome Sequencing Consortium 2011).

### Identification of centromere positions and pericentromeric regions

Centromere positions were determined with the sequence information provided by Gong *et al.* (2012). For chromosomes 4, 6, 9, 10, 11, and 12, the DM superscaffolds covering the centromere locations were identified from the major peaks in the CENH3 chromatin immunoprecipitation sequence read plots on the DM V2.1.10 PM sequences. Satellite repeat analysis was performed by searching for the repeats in the DM sequence at http://yh.genomics.org.cn/potato/search.jsp and by evaluating the repeat coverage through dot plot alignment of candidate DM sequences with the repeat sequence. In addition, centromere positions were also indirectly inferred from the marker density in RH UHD genetic map (van Os *et al.* 2006).

The revised physical positions of all of the Illumina Potato 8303 Infinium array SNPs, reported by Felcher *et al.* (2012) using their customized version (2.1.11) of potato reference PMs, were obtained for the latest version (4.03) of PMs (Table S3). Graphs depicting the progression of genetic distance and recombination rate *vs.* physical distance were calculated for all of the SNPs included in the current PMs and D84 and DRH genetic maps, using the MareyMap package (Rezvoy *et al.* 2007). The pericentromeric heterochromatin regions of the DM PMs were identified in these plots from the absence of genetic recombination between the SNP markers in such regions. In addition, AFLP markers from the marker-dense pericentromeric bins of the RH genetic maps were used to define heterochromatin boundaries in the PMs (Park *et al.* 2007), especially in cases where the genetic maps of Felcher *et al.* (2012) offered limited resolution.

### BAC assembly and comparison with PMs

A total of 96 DM BACs spanning scaffolding gaps on chromosome 4 were selected (using DM BAC-end hits; Potato Genome Sequencing Consortium 2011). The BACs were picked from the library and end-sequenced to verify correct selection. Eighty-two verified BACs were further processed and grouped into six normalized pools as well as a composite master pool containing all 82 BACs. Each of the six BAC pools was subjected to Roche 454 single-end shotgun sequencing and the master pool to 3-kb PE sequencing. Single-end data for each pool were combined with the PE data and were assembled together using the Newbler GSAssembler (Margulies *et al.* 2006). The sequences were deposited in the EBI Short Read Archive (accession number: ERP000934).

Candidate BAC scaffolds containing BAC-end sequences were identified with BLAST, filtering hits with a minimum match length of 400 bases and bit score exceeding 700 before manual curation. BAC scaffolds were matched to DM genomic superscaffolds with MUMmer (Kurtz *et al.* 2004). Matching regions were filtered to retain only matches longer than 1000bp with >97% identity. Data were expressed graphically with matches as edges and BAC end sequences, superscaffolds and BAC scaffolds as nodes using the graphical exchange format. Code was written in Python with the pygexf library and visualization performed with Gephi (http://www.gephi.org). In addition, BAC ends were linked by a BAC label as a node. Assemblies which linked superscaffolds with sequence data could then be readily observed as cycles containing a BAC label in the graph. BAC-oriented GFF files were generated and visualized with R.

## Results and Discussion

### DM genome assembly: a brief summary

The potato nuclear genome involved generation of ~96.6 Gb of raw sequence, which assembled into 66,254 “superscaffolds” comprising a net sequence assembly of 727 Mb, 117 Mb less than the estimated genome size of 844 Mb. Superscaffold length is inversely proportional to the numerical value in the name of each DM superscaffold (DMB), where the largest DMB (7.1 Mb) bears the ID “PGSC0003DMB000000001” and the smallest (100 bp) “PGSC0003DMB000066254.” Approximately 94% of the assembled genome is nongapped sequence and more than 90% of the genome (N_90_) is represented by 622 superscaffolds that are equal to or larger than 0.25 Mb. The anchoring strategy preferentially targeted the larger superscaffolds. At the time of publication 649 superscaffolds equaling 623 Mb (86%) of the assembled genome and 90% of the 39,031 estimated genes were anchored (Potato Genome Sequencing Consortium 2011). Draft PMs for the 12 chromosomes had been constructed but superscaffolds were mostly un-oriented. Since the original publication, continuous efforts have been made to perform further anchoring and orientation of the DM superscaffolds in order to generate the revised and improved genome PMs presented here (version 4.03).

### Genetic analysis of the mapping population

The DMDD mapping population was genotyped for AFLP, SSR, SNP, and DArT markers. Twenty two AFLP primer pairs (*Eco*RI/*Mse*I) amplified 213 detectable fragments. A total of 356 SSR loci were assayed. Of 2304 POPA SNPs and 7680 DArTs interrogated, 2160 and 2174 yielded genotype data, respectively. The compiled set of 4903 markers was screened for presence of polymorphism, data integrity, and concordance between parental and progeny genotypes, as well as meeting the missing data threshold (<20%) and other standard quality control checks. These data filtering and quality measures resulted in considerable reduction in the total number of markers used for linkage mapping to 2597, which comprised 187 AFLPs, 234 SSRs, 367 SNPs, and 1809 DArTs. After excluding co-segregating markers, we used a subset of 1864 uniquely segregating loci for linkage grouping; 1751 unique loci were incorporated into a combined parental linkage map with the 12 expected linkage groups, whereas the remaining 113 remained unmapped. The 12 chromosomal linkage groups span 936.2 cM with an average marker spacing of 0.54 cM per interval. The individual linkage groups ranged in size from 62.9 cM (Chr11) to 101.8 cM (Chr03). A combination of the use of previously mapped SSR markers (Veilleux *et al.* 1995; Milbourne *et al.* 1998; Feingold *et al.* 2005; Tang *et al.* 2008a; Ghislain *et al.* 2009) and other available resources such as the RH genetic map (Van Os *et al.* 2006), the RH WGP map (de Boer *et al.* 2012) and the tomato-EXPEN 2000 map (Fulton *et al.* 2002) allowed orientation and assignment of all 12 linkage groups to their respective chromosomes. Table 1 shows the summary statistics of linkage mapping in the DMDD cross.

**Table 1:** Distribution of 1751 markers comprising four different classes across the 12 chromosomes in the DMDD population, with the concomitant map and interval lengths (cM) for each chromosome

Chr***^a^***	Mapped Markers***^b^***	Map Length, cM	Interval Spacing, cM/interval***^c^***
01	201	93.0	0.46
02	221	77.4	0.35
03	134	101.8	0.77
04	143	99.7	0.70
05	107	64.1	0.61
06	134	70.5	0.53
07	108	67.1	0.63
08	176	67.8	0.39
09	152	87.9	0.58
10	144	68.9	0.48
11	108	62.9	0.59
12	123	75.2	0.62
All	1751	936.2	0.54

SSR, simple sequence repeat.

a Based on the SSRs mapped in previous studies and further confirmed by using *in silico* approaches.

b Excluding 718 co-segregating markers; when the segregation pattern of two or more markers was identical, only a single marker per set of identical markers was retained to generate the maps; 128 ungrouped markers (including 15 unassigned co-segregating markers) that did not fit any linkage group were also excluded.

c Calculated as the map length divided by the number of intervals (mapped markers minus 1, for “total” it is mapped markers minus 12).

Departure from Mendelian segregation has been observed frequently in potato crosses. Markers showing segregation distortion were not excluded from the mapping process and most could be mapped to their appropriate linkage groups. The frequency of segregation distortion was highly variable among different chromosomes with the most significant distorted regions observed on chromosomes 1 and 4. Previous potato mapping studies have also shown varying levels of segregation distortion (Gebhardt *et al.* 1991, Felcher *et al.* 2012). Figure S1 shows genome-wide distribution of levels of segregation distortion for all STS markers used in DMDD.

### Linkage map−based (direct) anchoring

**Figure 1:**
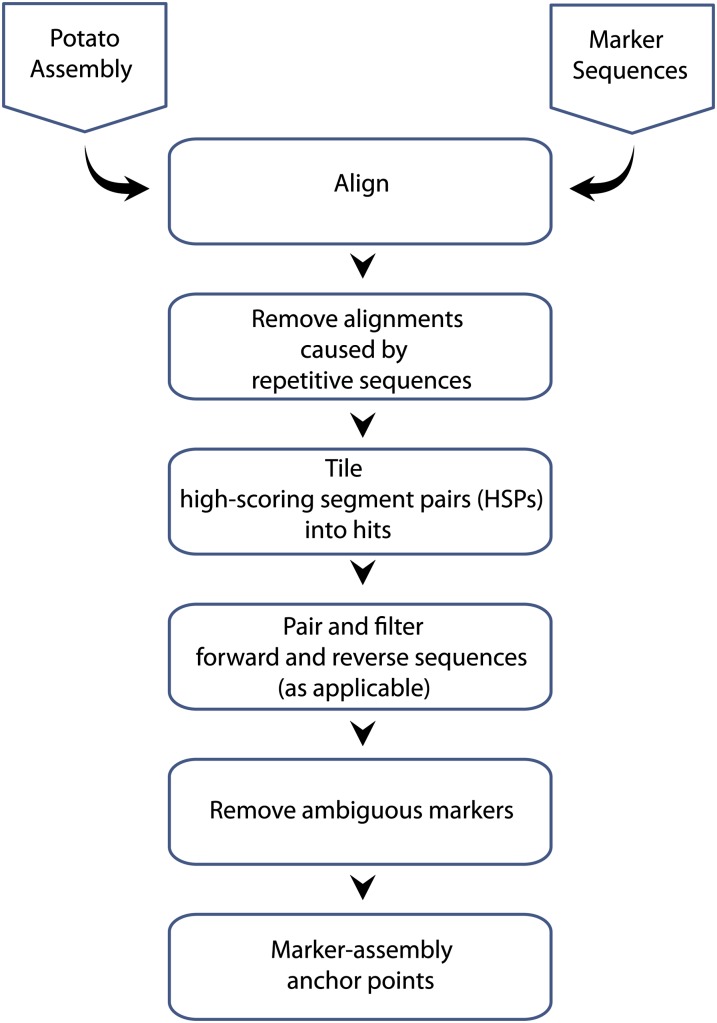
Pipeline for anchoring of markers to the potato genome assembly.

The linkage map of DMDD is predominantly composed of STS markers. The primary map-based anchoring strategy involved locating these sequence-based markers in the DM superscaffolds. SNPs and previously unpublished SSR markers (prefixed with “PM”) used in the DMDD linkage map were designed *a priori* against genome superscaffolds so their unique positions in the relevant superscaffolds were known. The positions of DArT and previously reported SSRs were determined using the bioinformatics alignment and filtering pipeline illustrated in Figure 1.

**Figure 2:**
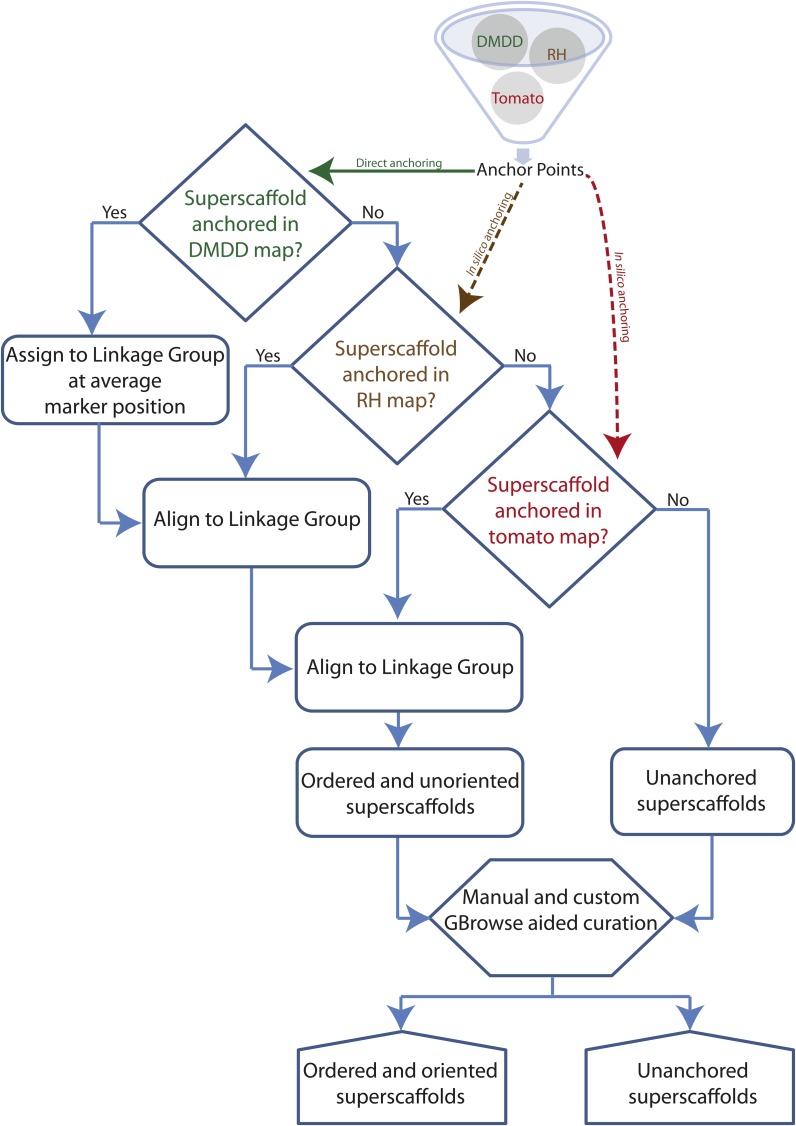
Step-wise linkage group assignment and ordering of DM superscaffolds using genetic-anchoring information successively from the DM, RH, and tomato genetic maps.

Co-segregating markers removed during linkage map construction were included in the anchoring process as such genetically redundant markers represent distinct, but physically linked sites in the genome. The complete set of STS markers was filtered for unique and unambiguous marker-assembly sequence alignments as described. The combined sequence and genetic map coordinates for these unique STS markers were used to assign and order superscaffolds for constructing a framework physical map. The integrated genetic and physical anchoring strategy is shown in Figure 2. Using this strategy, we anchored 1730 (1305 DArTs, 345 SNPs, and 80 SSRs) of the 2292 mapped, including co-segregating, STS markers to their unique positions on the DM superscaffolds. This approach anchored 54.2% (394 Mb) of the DM genome assembly arranged into 334 superscaffolds (Table 2). The proportion of genetic markers anchored on the genome sequence from each marker-category was 96% (SNPs), 28% (SSRs), and 76% (DArTs). Mapped AFLP fragments were not used in the anchoring process, due to a lack of sequence information. Table S2 contains genomic positions for all the STS markers used in the study. Genetic and physical coordinates for the DMDD mapped markers, including 718 co-segregating markers, are provided in Table S4.

**Table 2:** Anchoring statistics by chromosome for the three different physical maps, *de novo* (DM) and *in silico* (RH and tomato)

Chromosome	DM Map			RH Map			Tomato Map		
	DMB Anchored	Cumulative Length, Mb	No. of Markers*^a^*	DMB Anchored	Cumulative Length, Mb	No. of Markers	DMB Anchored	Cumulative Length, Mb	No. of Markers
01	39	45	162	69	80	208	43	41	271
02	35	43	175	35	43	120	33	40	233
03	19	24	108	28	27	73	41	45	194
04	34	47	138	51	57	168	40	39	174
05	20	27	74	33	45	137	25	30	112
06	29	34	108	44	46	119	34	34	133
07	26	24	89	35	39	122	32	31	136
08	32	32	152	24	23	57	40	32	129
09	27	28	109	34	33	91	40	39	136
10	31	38	106	34	44	102	26	32	110
11	20	26	113	36	38	110	22	26	116
12	22	26	72	47	52	164	26	28	109
Total	334	394	1406	470	527	1471	402	417	1853

DM, doubled monoploid reference clone; RH, RH89-039-16; DMB, DM superscaffold.

a Only markers mapped in DMDD and uniquely and reliably anchored to DM assembly are included.

### *In silico* approach−based (indirect) anchoring

The DMDD-based framework physical map was extended by integrating two additional sources of syntenic map data, from potato and tomato, respectively. First, superscaffolds anchored using the RH UHD genetic and physical maps (van Os *et al.* 2006; de Boer *et al.* 2012) were added. Second, 2,604 sequence-based markers from the tomato-EXPEN 2000 derived maps, which are closely collinear with potato (Tanksley *et al.* 1992; Fulton *et al.* 2002; The Tomato Genome Sequencing Consortium 2012), were used to add superscaffolds. In the case of RH, sequence anchoring was derived from the AFLP- and WGP-based hybrid RH physical map (de Boer *et al.* 2012) as well as by direct alignment of RH BAC end sequences and fully sequenced RH seed BACs to the DM sequence. In both cases, the (proxy) marker sequences were aligned to the DM assembly using BLAST, adopting stringent matching criteria. The results were processed into reliable genetic anchor points as described previously for the DM markers.

**Figure 3:**
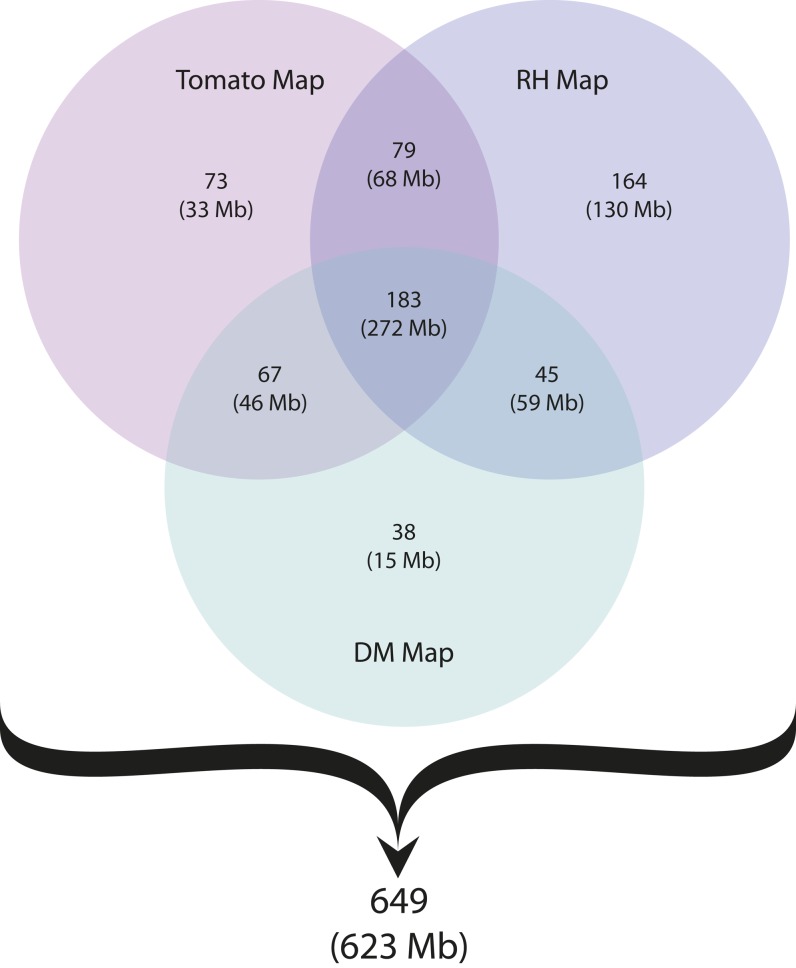
Summary of DM genome assembly anchoring using three different map resources. The number of uniquely and jointly anchored superscaffolds for each resource is given in the appropriate intersection. Cumulative size (Mb) of superscaffolds anchored in each category is shown in parenthesis. The total number of 649 anchored superscaffolds represents 623 Mb of the assembled DM potato genome. Figure updated from the Potato Genome Sequencing Consortium (2011).

The RH- and tomato-based *in silico* anchoring strategies independently anchored 470 (527 Mb, 72.5%) and 402 (417 Mb, 57.4%) superscaffolds, respectively (Table 2). Figure 3 shows the superscaffold anchoring summary for both the linkage (DM map) and the two *in silico* (RH and tomato maps) approaches. The total set of 649 superscaffolds anchored in at least one map was integrated hierarchically, starting with the DMDD-based framework map, placing additional superscaffolds using first the RH and then tomato assignment. The hierarchical ‘alignment’ of the maps is described below.

### Construction of chromosome-scale PMs

Following anchoring, the superscaffolds were ordered into chromosome-scale PMs in a hierarchical process using genetic, sequence and physical map data. The process is broken into two stages.

#### Stage I:

In the first stage the STS markers from the DMDD genetic map were aligned to the DM superscaffolds and used to construct the “backbone” PMs. Additional sequence-linked and sequence-based markers from the RH and tomato genetic maps were subsequently used to add superscaffolds into the DM backbone PMs (Figure 2). Superscaffolds that were anchored in multiple maps were used as reference points to align the genetic positions in the three different maps. Superscaffolds were added into ‘gaps’ in the backbone PMs where the positions indicated by the RH and tomato markers were in agreement with the positions initially established by the DMDD map data. The known set of chromosomal inversions on chromosomes 5, 6, 9, 10, 11, and 12 between potato and tomato (Tanksley *et al.* 1992; Iovene *et al.* 2008; Tang *et al.* 2008b) were taken into account when aligning the different genetic maps.

Generally the different anchoring approaches provided direct support for each other with respect to the relative placement of superscaffolds in the PM. With an optimal alignment/agreement for the superscaffold order among the three different maps used for anchoring, 294 of 374 superscaffolds present in at least one map were found to be in the same order as in the other two maps. In some instances, we observed that ordering of superscaffolds derived using RH and tomato maps was inconsistent with that obtained from the DMDD genetic map. The observed differences could be due to many factors, including technical issues such as mapping or assembly errors or biological properties, such as previously unknown structural differences between the compared genomes. However, given the size and complexity of the potato genome, it is encouraging that the placement of 79% of the superscaffolds was corroborated by the different methods employed.

Although superscaffolds were integrated into genomic blocks at this stage, they were unoriented and, due to the difficulty of aligning genetic maps, largely unordered at the chromosome level. To add, orient and refine the order of superscaffolds into an AGP for constructing chromosome-scale PMs, a separate process was implemented, as described below.

#### Stage II:

**Figure 4:**
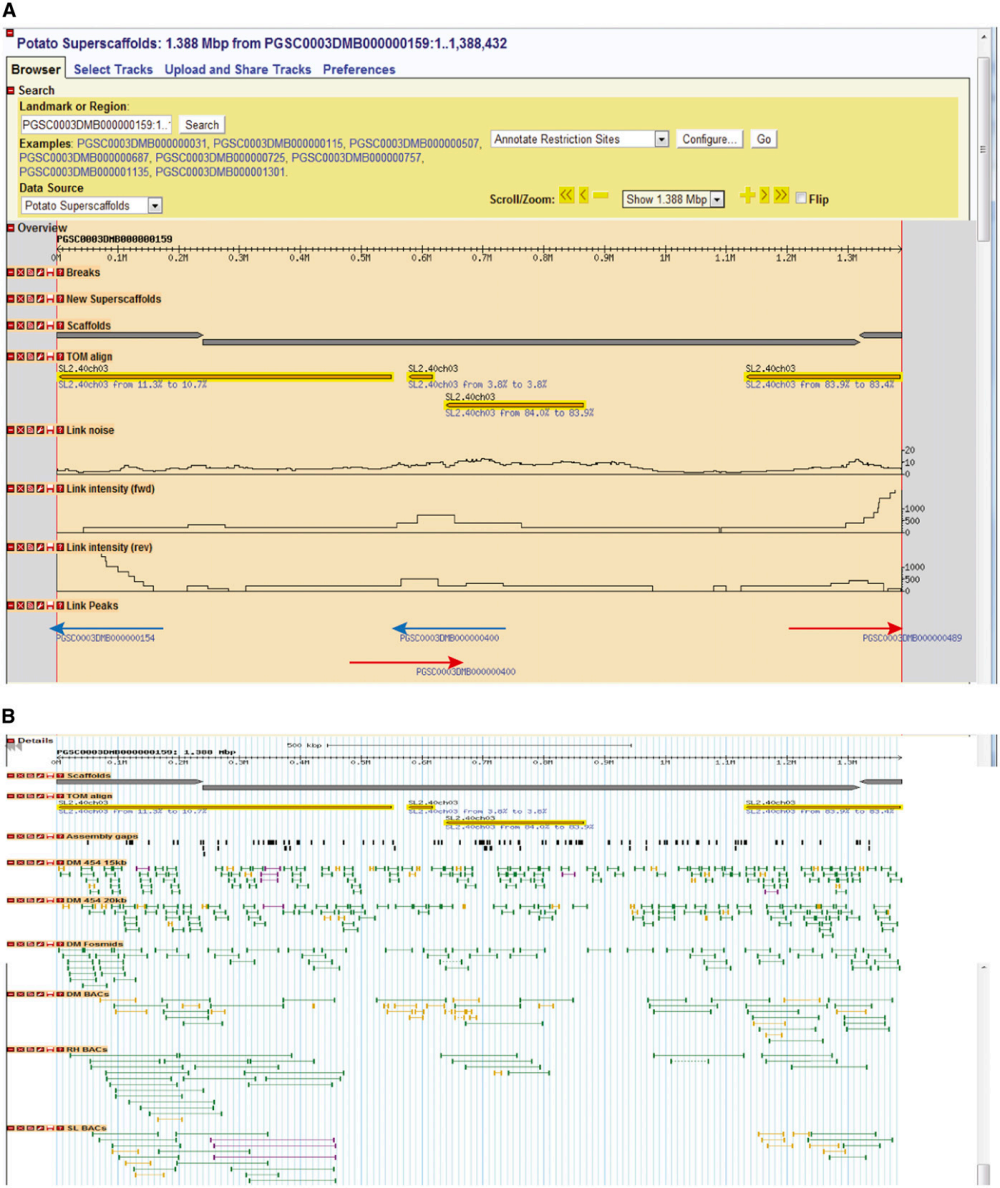
Depiction of “Link-peak” walk strategy taking superscaffold PGSC0003DMB000000159 as an example. (A) Custom GBrowse “Link-peak” intensity track features (shown as red and blue arrows) provided ordered navigation through superscaffolds using the aggregated PEMP. Link peaks to the right (red arrow) indicate “suggested path” downstream of the AGP, whereas those to the left (blue arrow) indicate converse. Reversal of this trend indicates a negative strand for the superscaffold in question. Traversing from one superscaffold to another by taking leads from these ‘Link-peak’ intensity tracks assisted in manually curating all 12 PMs. (B) Visualization of the underlying PEMP data.

To orient the DM superscaffolds, and to further refine the DMDD linkage map-based PMs, sequence and physical data from a variety of sources were combined as described in the *Materials and Methods* section and visualized on a standard GBrowse installation (Figure 4). Custom sequence features were created representing high scoring intersuperscaffold links, allowing the user to “click-and-walk” along the physical evidence from superscaffold to superscaffold in GBrowse. To aid this visualization, the processed RH WGP and tomato alignments, including the aligned sequence markers from the genetic maps used in stage I, were added to GBrowse as additional sequence feature tracks.

Using this integrated visualization tool, we performed three important types of manual improvements to the stage I PMs: (1) scaffolding links were used to provide the relative orientation of superscaffolds, (2) adjacent superscaffolds not previously included in the integrated genetic/physical map were added, and (3) errors in the assembly were identified. These manual improvements were mainly carried out for the euchromatic (gene-rich) regions and for the euchromatin/heterochromatin borders. In addition to orientating the majority of the anchored superscaffolds, the “link-peak” walk strategy combined with manual curation led to the incorporation of an additional 277 previously unanchored superscaffolds into the PMs.

During this process 67 chimeric superscaffolds were identified. Of these, 62, 3, and 2 superscaffolds were revealed to have one, two, and three misassembly locations, respectively, where false sequence joins had occurred. Many of these errors explained incongruities initially observed in the construction of the backbone PMs from the DMDD map (stage I). Chimeric superscaffolds were manually split and allocated to their respective positions in the PMs. For example, the sequence coordinates 1 to 1117982 bp of PGSC0003DMB000000002 were allocated to chromosome 4, whereas those from 1117983 to 6562806 bp were allocated to chromosome 5. These results further illustrate the utility of an integrated genetic and *in silico* anchoring based approach for refining and correcting genome assembly errors.

Included in the refinement process were dot plot alignments of DM chromosome PM sequences to pre-release and finished versions of the tomato genome sequence (The Tomato Genome Sequencing Consortium 2012). These alignments focused on the euchromatic regions and the adjacent heterochromatin border regions, where potato and tomato display homology in their sequences. The dot plot alignments to tomato made useful suggestions on how to place as yet unordered potato superscaffolds and superscaffold blocks, after which nearly always BAC end sequence links were identified in potato that confirmed the suggested orientation. Very occasionally, the potato PM description relied on the tomato alignment for placing potato sequence blocks in their presumed orientation, *e.g.*, from PGSC0003DMB000000729 to PGSC0003DMB000000835 at the top of chromosome 1 and from PGSC0003DMB000000692 to PGSC0003DMB000001163 in the south heterochromatin border on chromosome 8.

### Inversions with tomato

The potato-tomato dot plot alignments explained the discrepancies that were found between the potato and tomato genetic maps. In the euchromatic regions and the adjacent heterochromatin border regions we collected the sequence positions of the 19 largest paracentric inversions (with a length of at least 0.3 Mb), which are listed in Table S5 and also indicated in the DM PM figures. Newly identified were, among others, a tandem inversion with minor additional rearrangements on potato chromosome arm 1L, a nested inversion on 2L, and an arm inversion on 8S. Furthermore, the known arm inversions on 9S and 11L were found to be tandem inversions, with the second inversion being located in the heterochromatin border. The chromosomal rearrangements on 2L have also been described by Peters *et al.* (2012), who presented a scenario involving four structural conversions between potato and tomato. However, our dot plot sequence alignment for this region is less complex and shows a single, smaller inversion inside a larger inversion. This nested inversion model requires only two structural conversion steps and remains compatible with the cytogenetic results of Peters *et al.* (2012).

No paracentric inversions were identified on chromosome 3. However, on the short arm, the tomato sequence differs from the potato sequence by a 7.0-Mb insertion, which is located at position 2.4 Mb in the DM chromosome 3 PM, and which runs from 1.3 to 8.3 Mb in the tomato SL2.40 assembly. In its center, this tomato insert has 4.2 Mb of sequence that is largely devoid of genes (http://potato.plantbiology.msu.edu/), while the start and end regions align with gene-containing potato sequence segments from region 42.0 to 50.4 Mb on the south arm of chromosome 3. Although these data suggest a translocation of sequences across the centromere, further investigation is needed to exclude sequence assembly errors.

### Validation of link peak-based orientation strategy for chromosome 4

The strategy for PM construction and assembly correction was validated on chromosome 4 by targeted sequencing of 82 DM BAC clones that were selected to overlap candidate links as well as 10 of the 15 putative chimeric superscaffolds mapped to this chromosome. Thirty-one BAC clones could be assembled with contigs which spanned multiple superscaffolds and provided full coverage between the BAC end sequence matches to the superscaffolds, both validating the assembly and providing direct evidence for all 10 chimeric breakpoints. Seven of these sequenced BACs allow the inclusion of further superscaffolds that had not previously been assigned to a PM, and one provides evidence for a superscaffold that had been erroneously included.

**Figure 5:**
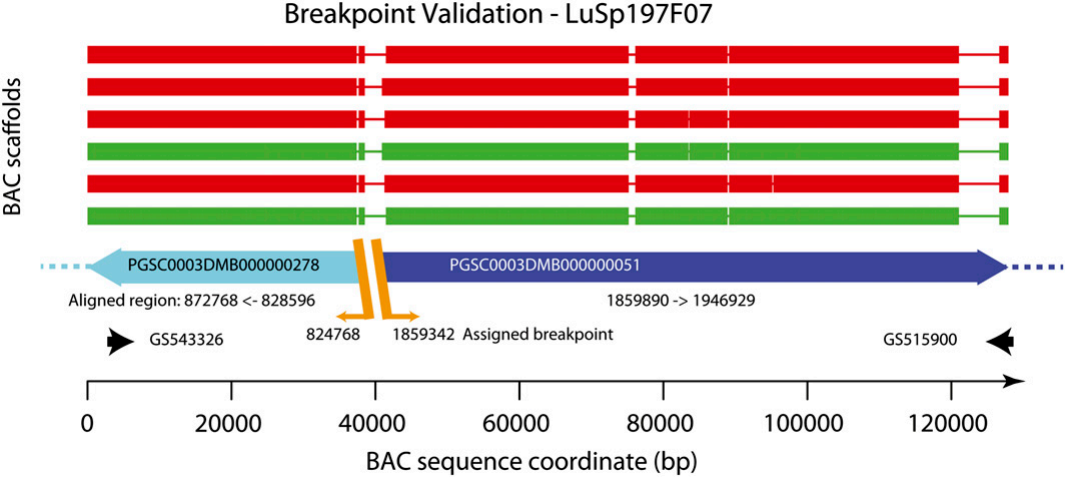
Assembled BAC sequence for LuSP197F07. Each scaffold assembly is derived from PE sequences of a combined pool of 82 DM BACs (spanning scaffolding gaps on chromosome 4) and single end sequence at greater read depth from one of the six subpools derived from the same BACs. The assemblies show a direct sequence running from PGSC0003DMB000000278 (− orientation, full length, cyan) through into PGSC0003DMB000000051 (+ orientation, blue) in accordance with the AGP and fully validating the decision to split PGSC00003DMB0000000278 at position 824768 and to split PGSC0003DMB000000051 at position 1859342 as indicated in the AGP file. Regions of good alignment (>98% identity, >1000 bases) are indicated as thick lines. Thin lines indicate no good alignment between the superscaffold and BAC sequences. The BAC end sequences are labeled with their Genbank IDs and are indicated at each end of the plot by black arrows. Breakpoints in the BAC sequences are indicated by orange diagonal lines and annotated with the assigned breakpoints coordinate from the AGP.

In addition to the complete assemblies described previously, most other clones could be assembled to a series of contigs which did not span multiple superscaffolds and which have not been included in the BAC pool assembly summary (Table S6). Details of the BAC analysis are given in the *Materials and Methods* section and a representative example validating a potential break-point in Chromosome 4 is illustrated in Figure 5. A list of putative erroneous superscaffold assembly locations (breakpoints), and the BACs which provide validation for them are given in Table S7.

### Demarcating centromeres and pericentromeric boundaries in the PMs

The putative centromere locations for 7 of the 12 potato chromosomes were identified in the PM sequences based on data published by Gong *et al.* 2012 (Table S8). Six centromere locations were identified from chromatin immunoprecipitated sequences. Of the seven published centromeric satellite repeat sequences (Gong *et al.* 2012), only the St24 repeat specific for the chromosome 1 centromere identified DM sequences with a high repeat copy number characteristic of centromeric regions. With the other six centromeric repeat sequences, we could not find reliable centromeric targets in the DM assembly because these sequences only identified locations with very few repeat copies, which sometimes occurred on a chromosome other than that expected from their designated centromeres.

**Figure 6:**
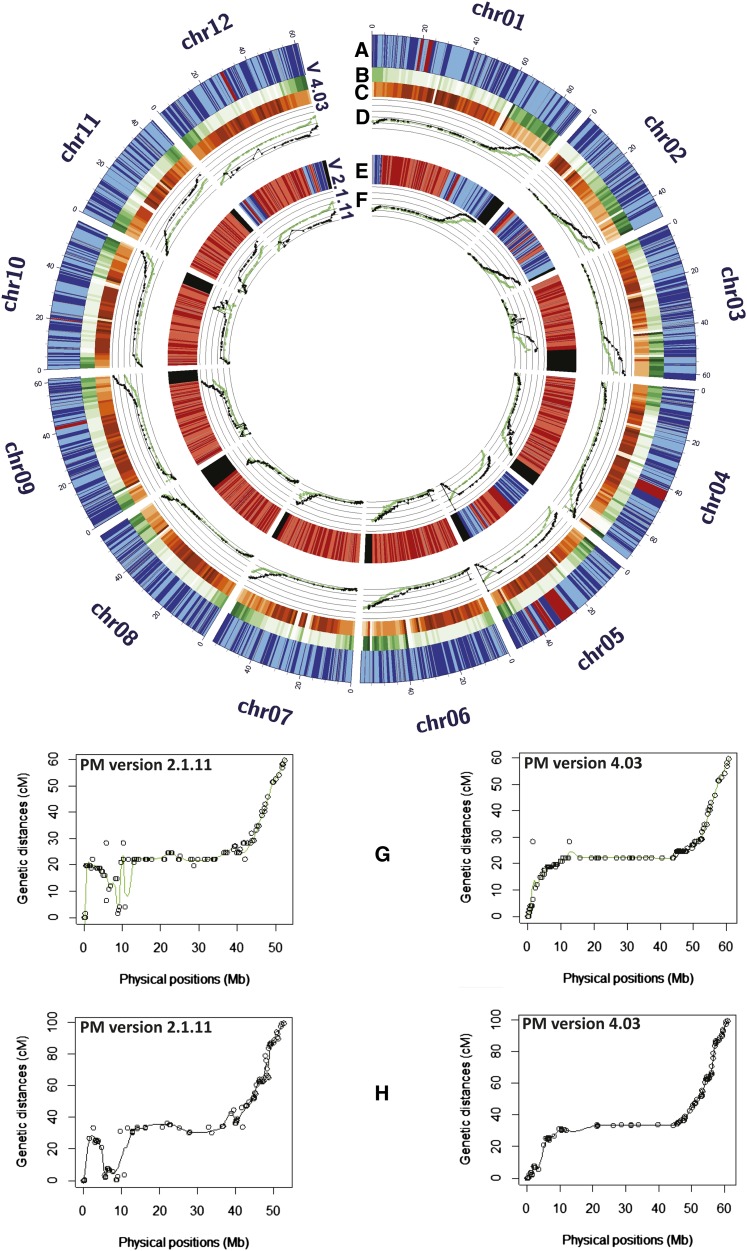
Enhanced accuracy of the current DM PMs. Panels A and E show anchoring of superscaffolds to the PM versions 4.03 and 2.1.11, respectively. Superscaffolds with known and unknown orientations are depicted in alternating shades of blue and red, respectively. Gaps in between the superscaffolds are marked in gray. Black areas in panel E represent unanchored superscaffolds (version 2.1.11) that were eventually anchored and ordered in PM version 4.03. Panels B and C show gene and repeat region densities, respectively, in 1 MB bins of PM version 4.03. Gene and repeat region densities ranges from 0 to >150 genes/MB and 0 to >900 repeats/MB, respectively. Panels D and F show the correspondence of the genetic maps (D84, green; DRH, black), adapted from Felcher *et al.* (2012), to PM versions 4.03 and 2.1.11, respectively. Graphs show the genetic (cM) positions plotted against the physical coordinates (Mb) for the SolCAP SNP markers; panels G (D84) and H (DRH) show elaborated examples of good correspondence from chromosome 9.

**Figure 7:**
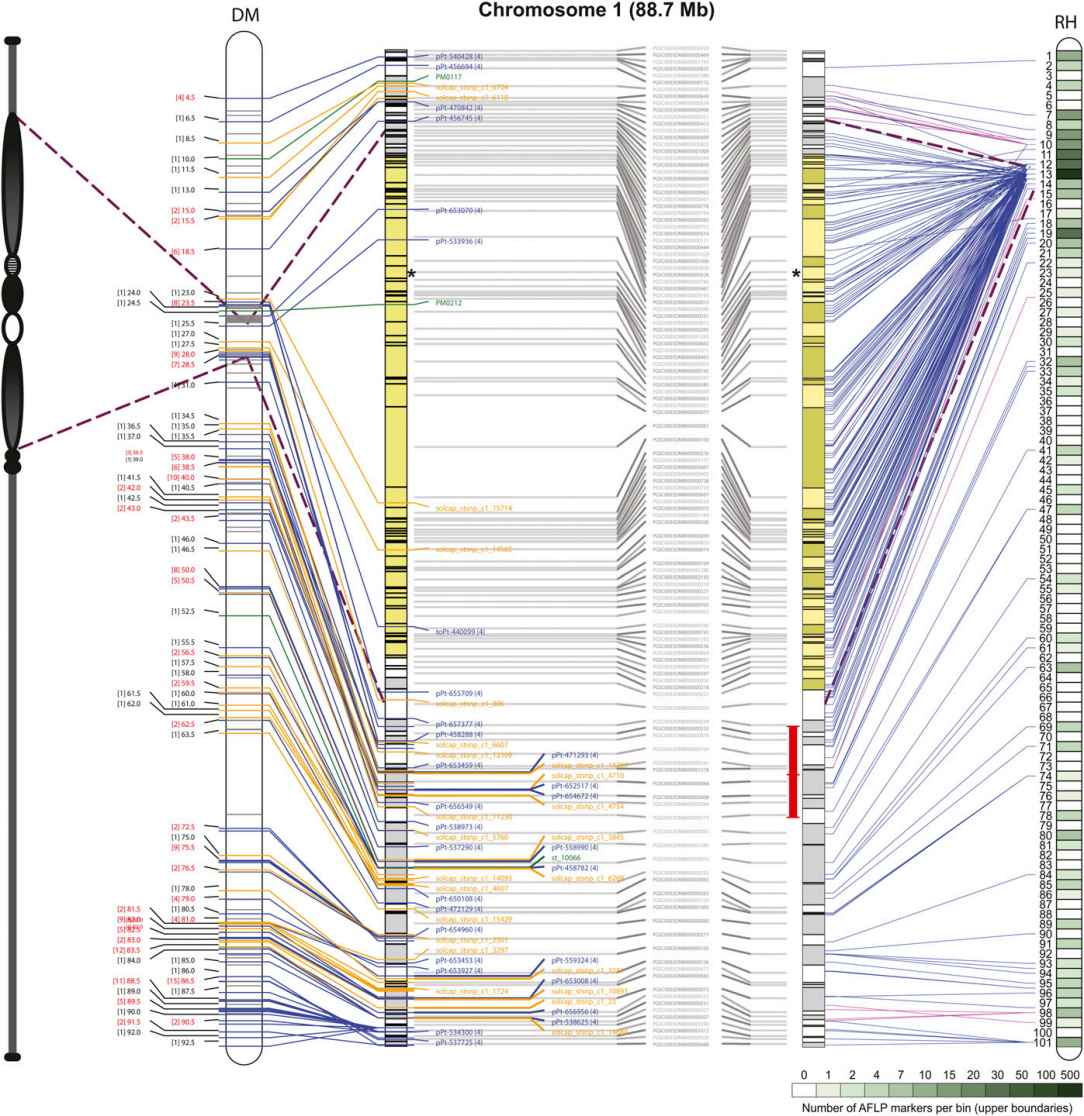
Illustration of the chromosome 1 PM integrated with the DM and RH genetic maps. STS and AFLP markers anchor sequence locations in the chromosome 1 PM to the DMDD and RH genetic maps, respectively. The AFLP marker positions in the PM were identified through sequence tag alignment of BAC clones from the RH WGP physical map. Superscaffolds comprising the PM are shown as alternating gray and white rectangular blocks. The layout of the PM for each of the genetic maps is shown separately but is identical with superscaffold IDs depicted in the middle. The pachytene idiogram is adapted from the potato reference genome publication (Potato Genome Sequencing Consortium 2011). The putative centromere region and pericentromeric/heterochromatic boundaries are demarcated by asterisk and dashed lines, respectively. Each DMDD marker type is color coded: blue = DArTs, yellow = SNPs, green = SSRs. Blue and magenta lines emerging from the RH genetic map represent AFLP anchors and the intensity of green color corresponds to the AFLP marker density per bin as reported by Van Os *et al.* (2006). Magenta lines represent AFLP markers with a relatively inaccurate mapping position on the RH genetic map, covering an interval of 5 or more bins. Regions in the central heterochromatin where superscaffold order and orientation are not completely resolved are indicated in yellow. Inversions with the tomato sequence are indicated with red interval bars.

Pericentromeric boundaries were deduced by comparing the SNP-based D84 and DRH genetic maps of Felcher *et al.* (2012) to the current version of PMs. For all chromosomes the typical pattern of distinctly reduced recombination in pericentromeric regions and increased varying recombination rates in euchromatic regions was observed (Figure 6). These patterns were used as the primary information source to demarcate putative pericentromeric regions in the PMs, and the boundaries of these regions were well supported, and where needed refined, by the RH genetic maps (van Os *et al.* 2006). Figure 7 and Figure S2 depict the centromere and pericentromeric locations in the PMs. The pachytene chromosome idiograms in these figures are adapted from Potato Genome Sequencing Consortium (2011).

### Current status of the reference PMs

The genome anchoring, ordering, and orienting process, as described previously, led to the joining of 951 genome superscaffolds, or nonchimeric segments thereof, into 144 larger, contiguous sequence blocks, and enabled construction of an AGP assembly for the reference DM potato genome. These chromosome-scale PMs, version 4.03, contain 93% (compared with 86%; Potato Genome Sequencing Consortium 2011) of the assembled genome comprising 674 Mb in 951 superscaffolds and include 37,482 (~96%) of the 39,031 predicted genes. A total of 938 superscaffolds (655 Mb or ~90% of the assembled genome sequence) are assigned absolute or relative orientation within the PMs, whereas the remaining 13 superscaffolds (19 Mb) are assigned with a random orientation. For 279 Mb of superscaffold sequence blocks from the heterochromatin, the exact chromosome position and absolute orientation could not be determined. These partially unordered regions are marked yellow in the PM figures (Figure 7 and Figure S2). No attempts were made to estimate gap sizes between the superscaffolds, and in the PM sequences all superscaffolds are separated from each other by a fixed gap sequence of 50,000 Ns. The N_90_ of the DM potato genome assembly is 0.25 Mb and contains 622 superscaffolds, of which 28 (equalling 17 Mb, ~2% of the assembled genome sequence) remain unanchored. The longest anchored superscaffold is 7.1 Mb (PGSC0003DMB000000001; chromosome 1) and the longest unanchored superscaffold (PGSC0003DMB000000064) is 2.2 Mb. The increase in average N_50_ from 1.5 Mb to 4.1 Mb in DM version 4.03 (Table 3) further supports the enhanced quality of the constructed PMs. The current version of the PMs/AGP is provided in Table S9 and includes the list of unanchored (chromosome 0) and chimeric superscaffolds.

**Table 3:** Improvements in DM PMs before and after execution of the link peak-based orientation strategy

Chr	Stage I*^a^*		Stage II*^b^*			
	DMB Anchored		DMB Anchored		DMB Oriented***^c^***	
	No (Size in Mb)	N_50_	No (Size in Mb)	N_50_	No (Size in Mb)	Percentage
01	83 (79.7)	1.7	123 (82.6)	2.6	121 (79.8)	96.6
02	51 (45.0)	1.3	68 (45.3)	2.2	68 (45.3)	100.0
03	53 (45.3)	1.6	103 (57.2)	4.3	103 (57.2)	100.0
04	73 (60.9)	1.2	120 (66.3)	2.9	119 (62.1)	93.7
05	41 (44.8)	1.7	52 (49.5)	2.9	47 (40.4)	81.6
06	63 (54.0)	1.4	90 (55.1)	2.7	90 (55.1)	100.0
07	52 (50.6)	1.8	78 (52.9)	7.2	78 (52.9)	100.0
08	51 (41.6)	1.2	91 (52.4)	4.9	91 (52.4)	100.0
09	61 (50.6)	1.2	86 (57.3)	8.3	85 (55.9)	97.7
10	50 (51.4)	1.5	77 (56.0)	4.1	74 (55.4)	99.0
11	35 (34.4)	1.4	60 (42.5)	5.7	60 (42.5)	100.0
12	61 (58.5)	1.5	77 (57.4)	1.9	76 (56.0)	97.7
Total	674 (616.8)*^d^*	1.5*^e^*	1025*^d^**^,^**^f^* (674.4)*^d^*	4.1*^e^*	1012*^d^**^,^**^f^* (655.1)*^d^*	97.2*^e^*

DM, doubled monoploid reference clone; PMs, pseudomolecules; DMB, DM superscaffold.

a Refers to the status of PMs before execution of the “Link-peak” walk strategy.

b Refers to the status of PMs after execution of the “Link-peak” walk strategy.

c Only attempted at stage II.

d Total.

e Average.

f Chimeric superscaffolds have been included more than once (net number of DMBs anchored = 951).

**Figure 8:**
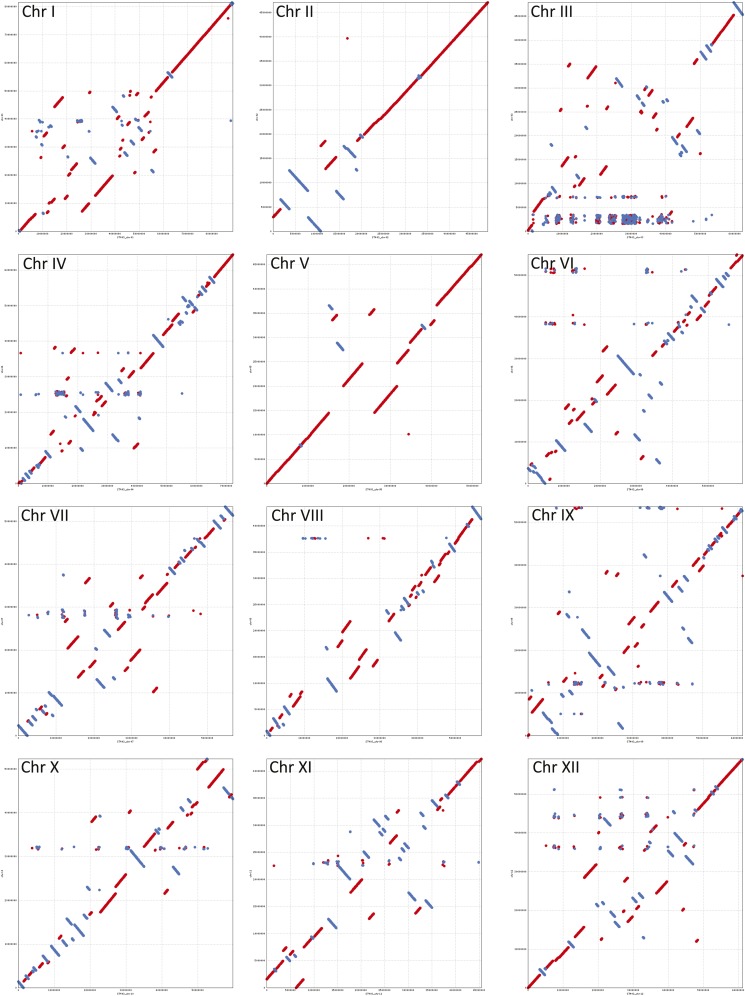
NUCmer sequence alignment dot plots for the twelve potato chromosomes using current (ver4.03, plotted on x-axis) and previous (ver2.1.11, plotted on y-axis) versions of DM PMs. Sequences aligned in forward and reverse orientations are represented by red and blue lines, respectively. Scaffold misplacements are shown as horizontal or vertical shifts in parts of the aligned blocks.

For visualizing the differences and improvements in the constructed PMs, we compared dot plots of the current PMs (ver 4.03) to the earlier version 2.1.11 (Figure 8). Superscaffold misplacements were apparent as horizontal or vertical shifts in parts of the alignments in all pairwise comparisons. The overall structural integrity of the constructed PMs is visible from the expected gradual transition from gene rich to gene poor regions which in turn are well complemented by the normal high repeat region density patterns in the pericentromeric locations gradually declining toward the gene rich euchromatic regions (Figure 6). The PMs along with integrated DMDD and RH genetic maps were visualized using DMAP as described in the *Materials and Methods* section. Figure 7 shows a representative illustration for chromosome 1 (chromosomes 2−12 are shown in Figure S2). Good correspondence between DMDD and RH genetic maps and the PMs was observed.

Although the DMDD map-based strategy was critical in providing the basic anchoring to the DM genome, it had its limitations. Certain superscaffolds lacked sufficient polymorphic STS markers for genomic anchoring and were possibly affected by homozygosity, segregation distortion or other issues (Figure S1). This mainly occurred in pericentromeric/heterochromatin regions (marked by dashed lines, Figure 7 and Figure S2), which generally displayed a sparse coverage with DMDD markers, possibly due to the customized marker design strategy that precluded the design of markers in highly repetitive, relatively gene poor regions. For example, SNPs were designed against coding regions using RNA-Seq data (Hamilton *et al.* 2011) and, thus, were mainly localized to gene-rich regions, which occupy a different “genomic space” to the gene-poor, high-repeat content regions (Figure 6). The DM-based “PM series” SSRs were designed from repeat-masked genome sequence to avoid placement in repetitive DNA. The DArT methodology also uses genome complexity reduction and has been shown to target the low copy fraction of a plant genome through judicious selection of certain restriction enzymes (Jaccoud *et al.* 2001). Thus, the unavoidable bias toward nonrepetitive sequences in the STS markers employed in the DMDD map resulted in many unanchored superscaffolds. This issue was resolved by using additional resources that we refer to as the *in silico* anchoring approach. For example, the large block of “orphaned” superscaffolds, not directly connected to the DMDD map, stretching from DMB 394 to DMB 705 (with the exception of DMBs 193, 15, 59, 100, and 200) on chromosome 1 (see Figure 7) was anchored by the evidence derived from the WGP/AFLP-based RH map and the tomato-EXPEN 2000 map and further extended by the “link-peak walk” strategy, illustrating the importance of the multi-layered anchoring approach adopted here.

Potato genomic resources are provided as tracks/features in the GBrowse for the DM genome (hosted at Spud DB site “http://potato.plantbiology.msu.edu/”). One such resource, widely adopted by the potato community, is the Illumina Potato 8303 SNP Infinium array (Felcher *et al.* 2012) released after our map was constructed. This SNP array was used by Felcher *et al.* (2012) to construct two genetic maps, both involving DM as the female parent. Although the homozygosity of DM precluded segregation of DM loci in these populations, they showed good congruence for most linkage groups to the prerelease version (a modified ver 2.1.10 latterly referred to as ver 2.1.11) of the DM PMs. Version 4.03 of the PMs provides an improved correspondence with the genetic maps of Felcher *et al.* (2012) (Figure 6). An updated annotation of the Illumina Potato 8303 SNP Infinium array is provided in Table S3. The DMDD genetic map and associated data files are available at http://solgenomics.net/, and include hyperlinks to the MSU Genome Browser. All of the supplementary data, wherever applicable, are available to download as GFF format files from Spud DB site “http://potato.plantbiology.msu.edu/”. The potato GBrowse including all of the hosted genomic resources/tracks/features have also been updated to the latest version (PM 4.03) of the DM PMs.

### Conclusions

The integrated genetic and physical reference map presented here comprising nearly 2500 markers, which are mostly STS, provides a platform for exploiting the potato reference genome. The most obvious and immediate application is the ability to position any sequence-based marker locus to a precise location in the DM genome. This will revolutionize trait analysis, although progress will be dependent on the complexity of the trait concerned, population size, replication and accuracy of phenotypic data and other factors that impinge on map resolution. Once mapped, the genome sequence around the locus can be used to design additional genetic markers for fine-scale mapping, and to identify putative candidate genes using the genome annotation. Such genes can be resequenced from informative plants showing phenotypic variation for the target trait. This ability to move directly from “map to genome to gene” will hasten the identification of genes responsible for traits. However, the automated annotation still includes many genes of “unknown function” and there are likely to be as yet unannotated genes in the genome sequence. Moreover, the DM genome represents only one haplotype in a species known to exhibit abundant sequence diversity.

The conversion of ~93% of the assembled genome sequence to well-structured, oriented and annotated PMs has made potato more amenable to modern genomic/genotyping approaches, such as genotyping-by-sequencing (Uitdewilligen *et al.* 2013). The clear and irreversible shift toward sequence based polymorphism in place of ‘fragment based’ markers will have the effect of augmenting centimorgan positions with genome sequence co-ordinates, providing a means for verifying the accuracy of mapping studies. The integrated DMDD map complements the published potato genome sequence and adds to a growing number of resources for genetic and genomic analyses.

The integrated map presented here and associated resources will help to alleviate many of the complicating aspects of potato as a genetic system. Potato is the most economically important crop where cultivars are highly heterozygous polyploids that suffer severe inbreeding depression on self-pollination. Such breeding systems make breeding and genetical studies difficult and cultivar development generally requires simultaneous recurrent selection for several traits over many years of evaluation. Introduction of traits that would make such crops more sustainable, *e.g.*, drought and salinity tolerance as well as nutrient use efficiency, will be targeted as we confront global climate change and dwindling natural resources (Levy *et al.* 2013). Moreover, attempts to convert the cross-pollinated tetraploid breeding system into an F_1_ hybrid diploid based scheme are also in progress (Lindhout *et al.* 2011). The isolation of genes coding for key traits, and characterization of their functional allelic diversity will be greatly facilitated by the resources provided in this study. A recent example is the identification of a gene largely responsible for the adaptation of Andean-derived potato germplasm to the longer day-lengths of temperate latitudes (Kloosterman *et al.* 2013).

The work presented here has generated a greatly improved ordering of the potato reference genome superscaffolds into chromosomal PMs. The reconfigured PMs and their links with genetic maps provide a major new resource for the research community. They form the basis by which geneticists can identify genes underlying important traits and through which comparative genomics can be further exploited in diversity assessment, phylogenetic inference, and plant breeding.

## Supplementary Material

Supporting Information

## References

[bib1] Buntjer J. B., 1999 Cross Checker, Vol. 291 Department of Plant Breeding, Wageningen University and Research Centre, Wageningen

[bib2] Creste S. Neto A. T. Figueira A., 2001 Detection of single sequence repeat polymorphisms in denaturing polyacrylamide sequencing gels by silver staining. Plant Mol. Biol. Rep. 19: 299–306

[bib3] de Boer J. M. Borm T. J. A. Jesse T. Brugmans B. Wiggers-Perebolte L., 2012 A hybrid BAC physical map of potato: a framework for sequencing a heterozygous genome (vol 12, 594, 2011). BMC Genomics 13: 423 10.1186/1471-2164-12-594 PMC3261212 22142254

[bib4] Fan J. B. Oliphant A. Shen R. Kermani B. G. Garcia F., 2003 Highly parallel SNP genotyping. Cold Spring Harb. Symp. Quant. Biol. 68: 69–78 10.1101/sqb.2003.68.69 15338605

[bib5] Feingold S. Lloyd J. Norero N. Bonierbale M. Lorenzen J., 2005 Mapping and characterization of new EST-derived microsatellites for potato (*Solanum tuberosum* L.). Theor. Appl. Genet. 111: 456–466 15942755 10.1007/s00122-005-2028-2

[bib6] Felcher K. J. Coombs J. J. Massa A. N. Hansey C. N. Hamilton J. P., 2012 Integration of two diploid potato linkage maps with the potato genome sequence. PLoS ONE 7: e36347 22558443 10.1371/journal.pone.0036347 PMC3338666

[bib7] Fulton T. M. Van der Hoeven R. Eannetta N. T. Tanksley S. D., 2002 Identification, analysis, and utilization of conserved ortholog set markers for comparative genomics in higher plants. Plant Cell 14: 1457–1467 12119367 10.1105/tpc.010479 PMC150699

[bib8] Gebhardt C. Ritter E. Barone A. Debener T. Walkemeier B., 1991 RFLP maps of potato and their alignment with the homoeologous tomato genome. Theor. Appl. Genet. 83: 49–57 24202256 10.1007/BF00229225

[bib9] Ghislain M. Núñez J. Herrera M. R. Pignataro J. Guzman F., 2009 Robust and highly informative microsatellite-based genetic identity kit for potato. Mol. Breed. 23: 377–388

[bib10] Gong Z. Wu Y. Koblízková A. Torres G. A. Wang K., 2012 Repeatless and repeat-based centromeres in potato: implications for centromere evolution. Plant Cell 24: 3559–3574 22968715 10.1105/tpc.112.100511 PMC3480287

[bib11] Green E. D. Green P., 1991 Sequence-tagged site (STS) content mapping of human chromosomes: theoretical considerations and early experiences. PCR Methods Appl. 1: 77–90 1842934 10.1101/gr.1.2.77

[bib12] Hamilton J. P. Buell C. R., 2012 Advances in plant genome sequencing. Plant J. 70: 177–190 22449051 10.1111/j.1365-313X.2012.04894.x

[bib13] Hamilton J. P. Hansey C. N. Whitty B. R. Stoffel K. Massa A. N., 2011 Single nucleotide polymorphism discovery in elite North American potato germplasm. BMC Genomics 12: 302 21658273 10.1186/1471-2164-12-302 PMC3128068

[bib14] Herrera M. R. Ghislain M., 2000 Molecular Biology Laboratory Protocols: Plant Genotyping, Ed. 3 Crop Improvement and Genetic Resources Department, International Potato Center (CIP), Lima, Peru

[bib15] International Rice Genome Sequencing Project, 2005 The map-based sequence of the rice genome. Nature 436: 793–800 16100779 10.1038/nature03895

[bib16] Iovene M. Wielgus S. M. Simon P. W. Buell C. R. Jiang J. M., 2008 Chromatin structure and physical mapping of chromosome 6 of potato and comparative analyses with tomato. Genetics 180: 1307–1317 18791232 10.1534/genetics.108.093179 PMC2581936

[bib17] Istrail S. Sutton G. G. Florea L. Halpern A. L. Mobarry C. M., 2004 Whole-genome shotgun assembly and comparison of human genome assemblies. Proc. Natl. Acad. Sci. USA 101: 1916–1921 14769938 10.1073/pnas.0307971100 PMC357027

[bib18] Jaccoud D. Peng K. Feinstein D. Kilian A., 2001 Diversity arrays: a solid state technology for sequence information independent genotyping. Nucleic Acids Res. 29: E25 11160945 10.1093/nar/29.4.e25 PMC29632

[bib19] Kloosterman B. Abelenda J. A. Gomez M. M. C. Oortwijn M. de Boer J. M., 2013 Naturally occurring allele diversity allows potato cultivation in northern latitudes. Nature 495: 246–250 23467094 10.1038/nature11912

[bib20] Kurtz S. Phillippy A. Delcher A. L. Smoot M. Shumway M., 2004 Versatile and open software for comparing large genomes. Genome Biol. 5: R12 14759262 10.1186/gb-2004-5-2-r12 PMC395750

[bib21] Levy D. Coleman W. K. Veilleux R. E., 2013 Adaptation of potato to water shortage: irrigation management and enhancement of tolerance to drought and salinity. Am. J. Potato Res. 90: 186–206

[bib22] Lindhout P. Meijer D. Schotte T. Hutten R. C. B. Visser R. G. F., 2011 Towards F 1 hybrid seed potato breeding. Potato Res. 54: 301–312

[bib23] Margulies M. Egholm M. Altman W. E. Attiya S. Bader J. S., 2006 Genome sequencing in microfabricated high-density picolitre reactors (vol 437, pg 376, 2005). Nature 441: 120–120 10.1038/nature03959 PMC1464427 16056220

[bib24] Milbourne D. Meyer R. C. Collins A. J. Ramsay L. D. Gebhardt C., 1998 Isolation, characterisation and mapping of simple sequence repeat loci in potato. Mol. Gen. Genet. 259: 233–245 9749666 10.1007/s004380050809

[bib25] Miller J. R. Koren S. Sutton G., 2010 Assembly algorithms for next-generation sequencing data. Genomics 95: 315–327 20211242 10.1016/j.ygeno.2010.03.001 PMC2874646

[bib26] Ning Z. M. Cox A. J. Mullikin J. C., 2001 SSAHA: a fast search method for large DNA databases. Genome Res. 11: 1725–1729 11591649 10.1101/gr.194201 PMC311141

[bib27] Ovchinnikova A. Krylova E. Gavrilenko T. Smekalova T. Zhuk M., 2011 Taxonomy of cultivated potatoes (*Solanum* section *Petota*: Solanaceae). Bot. J. Linn. Soc. 165: 107–155

[bib28] Park T. H. Kim J. B. Hutten R. C. van Eck H. J. Jacobsen E., 2007 Genetic positioning of centromeres using half-tetrad analysis in a 4x-2x cross population of potato. Genetics 176: 85–94 17339217 10.1534/genetics.107.070870 PMC1893073

[bib29] Paz M. M. Veilleux R. E., 1999 Influence of culture medium and in vitro conditions on shoot regeneration in *Solanum phureja* monoploids and fertility of regenerated doubled monoploids. Plant Breed. 118: 53–57

[bib30] Peters S. A. Bargsten J. W. Szinay D. van de Belt J. Visser R. G., 2012 Structural homology in the Solanaceae: analysis of genomic regions in support of synteny studies in tomato, potato and pepper. Plant J. 71: 602–614 22463056 10.1111/j.1365-313X.2012.05012.x

[bib31] Potato Genome Sequencing Consortium, 2011 Genome sequence and analysis of the tuber crop potato. Nature 475: 189–195 21743474 10.1038/nature10158

[bib32] Rezvoy C. M. Charif D. Gue’guen L. Marais G. A. B., 2007 MareyMap: an R-based tool with graphical interface for estimating recombination rates. Bioinformatics 23: 2188–2189 17586550 10.1093/bioinformatics/btm315

[bib33] Sliwka J. Jakuczun H. Chmielarz M. Hara-Skrzypiec A. Tomczyn’ska I., 2012 A resistance gene against potato late blight originating from Solanum x michoacanum maps to potato chromosome VII. Theor. Appl. Genet. 124: 397–406 21987281 10.1007/s00122-011-1715-4 PMC3258389

[bib34] Spooner D. M. Núñez J. Trujillo G. Herrera M. D. Guzmán F., 2007 Extensive simple sequence repeat genotyping of potato landraces supports a major reevaluation of their gene pool structure and classification. Proc. Natl. Acad. Sci. USA 104: 19398–19403 18042704 10.1073/pnas.0709796104 PMC2148301

[bib35] Tang J. F. Baldwin S. J. Jacobs J. M. E. van der Linden C. G. Voorrips R. E., 2008 a Large-scale identification of polymorphic microsatellites using an in silico approach. BMC Bioinformatics 9: 374 18793407 10.1186/1471-2105-9-374 PMC2562394

[bib36] Tang X. Szinay D. Lang C. Ramanna M. S. van der Vossen E. A., 2008 b Cross-species bacterial artificial chromosome-fluorescence *in situ* hybridization painting of the tomato and potato chromosome 6 reveals undescribed chromosomal rearrangements. Genetics 180: 1319–1328 18791231 10.1534/genetics.108.093211 PMC2581937

[bib37] Tang X. de Boer J. M. van Eck H. J. Bachem C. Visser R. G., 2009 Assignment of genetic linkage maps to diploid *Solanum tuberosum* pachytene chromosomes by BAC-FISH technology. Chromosome Res. 17: 899–915 19774472 10.1007/s10577-009-9077-3 PMC2776164

[bib38] Tanksley S. D. Ganal M. W. Prince J. P. Devicente M. C. Bonierbale M. W., 1992 High-density molecular linkage maps of the tomato and potato genomes. Genetics 132: 1141–1160 1360934 10.1093/genetics/132.4.1141 PMC1205235

[bib39] The Arabidopsis Genome Initiative, 2000 Analysis of the genome sequence of the flowering plant *Arabidopsis thaliana*. Nature 408: 796–815 11130711 10.1038/35048692

[bib40] The French-Italian Public Consortium for Grapevine Genome Characterization, 2007 The grapevine genome sequence suggests ancestral hexaploidization in major angiosperm phyla. Nature 449: 463–467 17721507 10.1038/nature06148

[bib41] The Tomato Genome Sequencing Consortium, 2012 The tomato genome sequence provides insights into fleshy fruit evolution. Nature 485: 635–641 22660326 10.1038/nature11119 PMC3378239

[bib42] Uitdewilligen J. G. Wolters A. M. D’hoop B. B. Borm T. J. Visser R. G., 2013 A next-generation sequencing method for genotyping-by-sequencing of highly heterozygous autotetraploid potato. PLoS ONE 8: e62355 23667470 10.1371/journal.pone.0062355 PMC3648547

[bib43] Van Ooijen J. W., 2006 Joinmap 4: Software for the Calculation of Genetic Linkage Maps. Kyazma B. V., Wageningen, The Netherlands

[bib44] van Os H. Andrzejewski S. Bakker E. Barrena I. Bryan G. J., 2006 Construction of a 10,000-marker ultradense genetic recombination map of potato: Providing a framework for accelerated gene isolation and a genomewide physical map. Genetics 173: 1075–1087 16582432 10.1534/genetics.106.055871 PMC1526527

[bib45] Veilleux R. E. Shen L. Y. Paz M. M., 1995 Analysis of the genetic composition of anther-derived potato by randomly amplified polymorphic DNA and simple sequence repeats. Genome 38: 1153–1162 8654912 10.1139/g95-153

[bib46] Vos P. Hogers R. Bleeker M. Reijans M. Vandelee T., 1995 AFLP: a new technique for DNA-fingerprinting. Nucleic Acids Res. 23: 4407–4414 7501463 10.1093/nar/23.21.4407 PMC307397

[bib47] Wenzl P. Carling J. Kudrna D. Jaccoud D. Huttner E., 2004 Diversity arrays technology (DArT) for whole-genome profiling of barley. Proc. Natl. Acad. Sci. USA 101: 9915–9920 15192146 10.1073/pnas.0401076101 PMC470773

