# Supplementary material for: Construction of Reference Chromosome-Scale Pseudomolecules for Potato: Integrating the Potato Genome with Genetic and Physical Maps
Source: G3 (Bethesda). 2013 Nov 1;3(11):2031–47. doi: 10.1534/g3.113.007153 (PMC3815063; doi:10.1534/g3.113.007153)
Supplement: Supporting Information [file supp_3_11_2031__index.html]

Construction of Reference Chromosome-Scale Pseudomolecules for Potato: Integrating the Potato Genome with Genetic and Physical Maps — Supporting Information 

# Construction of Reference Chromosome-Scale Pseudomolecules for Potato: Integrating the Potato Genome with Genetic and Physical Maps

## Supporting Information for Sharma *et al.*, 2013

**Files in this Data Supplement:**

- Supporting Information - Figures S1-S2 and Tables S1-S9 (PDF, 5 MB)
- Figure S1 - Genome-wide patterns of marker segregation distortion in DMDD population for 1830 STS markers from different segregation categories plotted as a function of Chi-square value (y-axis) against marker physical position (x-axis) on each of the 12 potato chromosomes. (PDF, 983 KB)
- Figure S2 - Illustration of the chromosome 2 - 12 pseudomolecules (PMs) integrated with the DM and RH genetic maps. (PDF, 5 MB)
- Table S1 - Details of (A) Simple sequence repeat (SSR), (B) Single nucleotide polymorphism (SNP) and (C) Amplified fragment length polymorphism (AFLP) markers employed in DMDD genotyping. (.xls, 573 KB)
- Table S2 - Location of sequence-tagged site (STS) markers employed in DMDD genotyping on the DM version 3 superscaffolds and DM version 4.03 pseudomolecules. (.xls, 2 MB)
- Table S3 - Revised annotation details for the Infinium 8.3k Potato Array SNPs (Felcher *et al.* 2012) on DM version 4.03 pseudomolecules. (.xls, 2 MB)
- Table S4 - Genetic and physical locations of STS markers (DArTs, SSRs and SNPs) mapped in DMDD and anchored in DM version 4.03 pseudomolecules. (.xls, 458 KB)
- Table S5 - Paracentric inversions between potato and tomato chromosomes detected by dot plot alignments between the chromosome pseudomolecules V4.03 of potato line DM and V2.40 of tomato cv. 'Heinz 1706'. (.xls, 37 KB)
- Table S6 - Summary of six BAC pools sequence assembly data comprising 82 DM BAC clones used for validating link peak-based orientation strategy for chromosome 4. (.xls, 32 KB)
- Table S7 - BAC pool assembly and validation details for chromosome 4 pseudomolecule version 4.03. (.xls, 60 KB)
- Table S8 - Centromere localization in DM V3 sequence assembly. (.xls, 33 KB)
- Table S9 - Accessioned Golden Path (AGP) for the reference DM chromosome-scale pseudomolecules version 4.03. File also includes revised annotation details for potato genes and repeat regions (Potato Genome Sequencing Consortium 2011) and a list of chimeric superscaffolds. (.xls, 75 MB)
