# Supplementary material for: Construction of Reference Chromosome-Scale Pseudomolecules for Potato: Integrating the Potato Genome with Genetic and Physical Maps
Source: G3 (Bethesda). 2013 Nov 1;3(11):2031–47. doi: 10.1534/g3.113.007153 (PMC3815063; doi:10.1534/g3.113.007153)
Supplement: Supporting Information [file supp_g3.113.007153_007153SI.pdf]

G3: Genes | Genomes | Genetics

Construction of reference chromosome-scale pseudomolecules for potato: Integrating the potato genome with genetic and physical maps

Sanjeev Kumar Sharma[*,1], Daniel Bolser[§,1,2], Jan de Boer[†], Mads Sønderkær[‡], Walter Amoros[**], Martin Federico Carboni[§§], Juan Martín D'Ambrosio[§§], German de la Cruz[††], Alex Di Genova[‡‡], David S. Douches[***], Maria Eguiluz[§§§], Xiao Guo[†††], Frank Guzman[§§§,3], Christine A. Hackett[‡‡‡], John P. Hamilton[****], Guangcun Li[†††], Ying Li[§§§§], Roberto Lozano[§§§], Alejandro Maass[‡‡], David Marshall[††††], Diana Martinez[§§§], Karen McLean[*], Nilo Mejía[‡‡‡‡], Linda Milne[††††], Susan Munive[**], Istvan Nagy[*****,4], Olga Ponce[§§§], Manuel Ramirez[§§§], Reinhard Simon[**], Susan J. Thomson[§§§§§], Yerisf Torres[§§§], Robbie Waugh[*], Zhonghua Zhang[§§§§], Sanwen Huang[§§§§], Richard G. F. Visser[†], Christian W. B. Bachem[†], Boris Sagredo[†††††], Sergio E. Feingold[§§], Gisella Orjeda[§§§], Richard E. Veilleux[‡‡‡‡‡], Merideth Bonierbale[**], Jeanne M. E. Jacobs[§§§§§], Dan Milbourne[*****], David Michael Alan Martin[§], Glenn J. Bryan[*,5]

[*]Cell and Molecular Sciences, The James Hutton Institute, Dundee DD2 5DA, United Kingdom
[§]Division of Biological Chemistry and Drug Discovery, College of Life Sciences, University of Dundee, Dundee DD1 5EH, United Kingdom
[†]Laboratory of Plant Breeding, Department of Plant Sciences, Wageningen-UR, Wageningen, The Netherlands
[‡]Department of Biotechnology, Chemistry and Environmental Engineering, Aalborg University, Aalborg, Denmark
[**]International Potato Center (CIP), Lima 12, Peru
[§§]Laboratorio de Agrobiotecnología, Instituto Nacional de Tecnología Agropecuaria (INTA) cc276 (7620) Balcarce, Argentina
[††]Laboratorio de Genética y Biotecnología Vegetal, Universidad Nacional San Cristobal de Huamanga, Ayacucho, Perú
[‡‡]Mathomics, Centro de Regulación Genómica & Centro de Modelamiento Matemático, Universidad de Chile, Santiago, Chile
[***]Department of Crop and Soil Sciences, Michigan State University, Michigan, United States of America
[§§§]Genomics Research Unit, Facultad de Ciencias, Universidad Peruana Cayetano Heredia, Lima 31, Peru
[†††]Institute of Vegetables, Shandong Academy of Agricultural Sciences, Jinan 250100, China
[‡‡‡]Biomathematics and Statistics Scotland, Dundee DD2 5DA, United Kingdom
[****]Department of Plant Biology, Michigan State University, Michigan, United States of America
[§§§§]Institute of Vegetables and Flowers, Chinese Academy of Agricultural Sciences, Beijing 100081, China
[††††]Information and Computational Sciences, The James Hutton Institute, Dundee DD2 5DA, United Kingdom
[‡‡‡‡]INIA-La Platina, Santiago, Chile
[*****]Crops Environment and Land Use Programme, Teagasc, Carlow, Ireland
[§§§§§]The New Zealand Institute for Plant & Food Research Ltd., Christchurch 8120, New Zealand
[†††††]INIA-Rayentué, Rengo, Chile
[‡‡‡‡‡]Department of Horticulture, Virginia Tech, Virginia, United States of America

Footnotes:
[1]S. K. Sharma and D. Bolser contributed equally to this work
[2]Current Address: The EMBL-European Bioinformatics Institute, Wellcome Trust Genome Campus, Cambridge CB10 1SD, United Kingdom
[3]Current Address: Departamento de Genética, Universidade Federal do Rio Grande do Sul - UFRGS, Rio Grande do Sul, Brazil
[4]Current Address: Department of Molecular Biology and Genetics, Aarhus University, Slagelse DK-4200, Denmark
[5]Corresponding Author

DOI: 10.1534/g3.113.007153

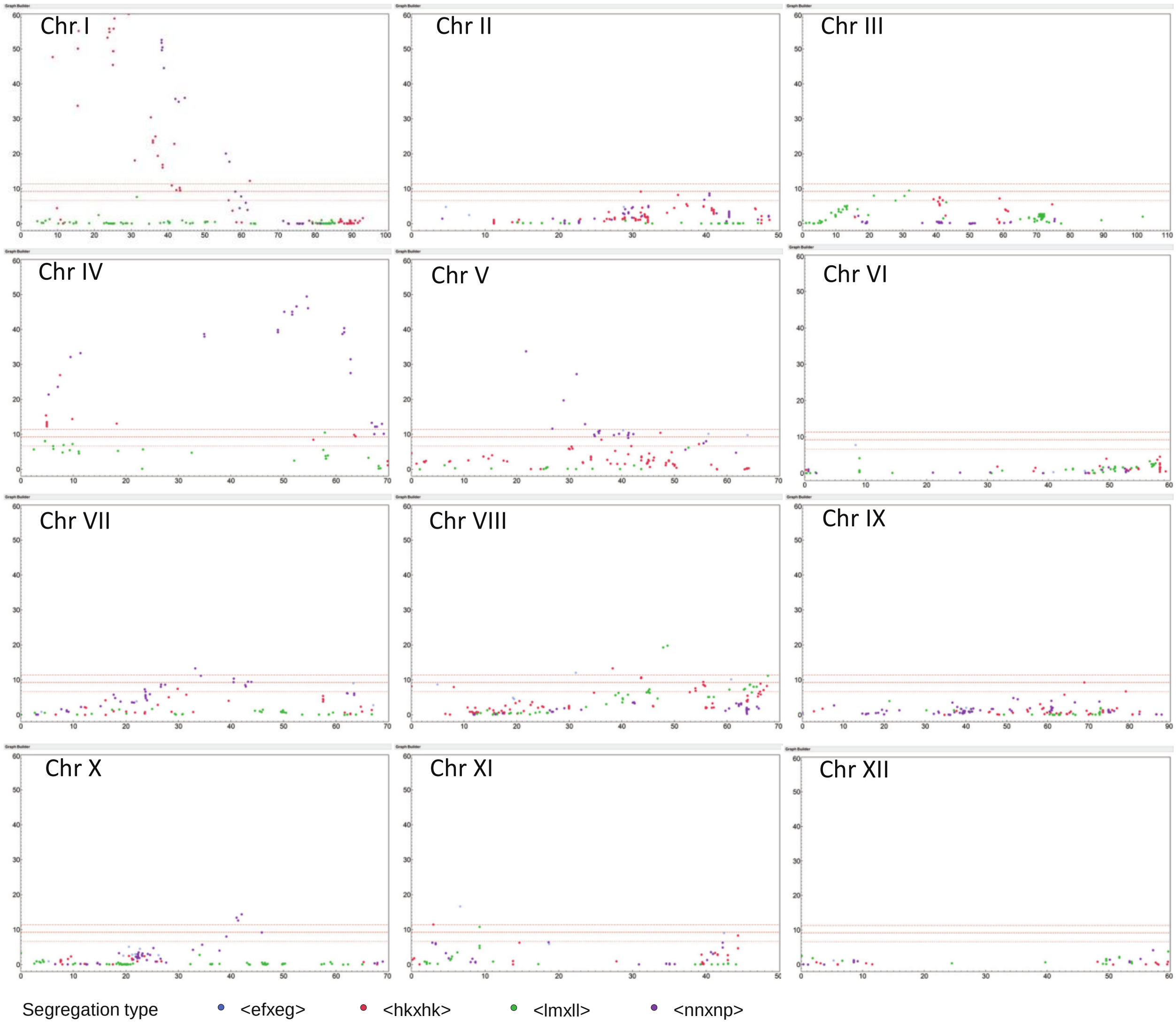

Figure S1 Genome-wide patterns of marker segregation distortion in DMDD population for 1830 STS markers from different segregation categories plotted as a function of Chi-square value (y-axis) against marker physical position (x-axis) on each of the 12 potato chromosomes. Dotted, dashed and dotted-dashed lines represent Chi-square significance values at p = 0.01 for marker segregation categories with two, three and four genotypic classes, respectively.

Chromosome 2 (48.6 Mb)

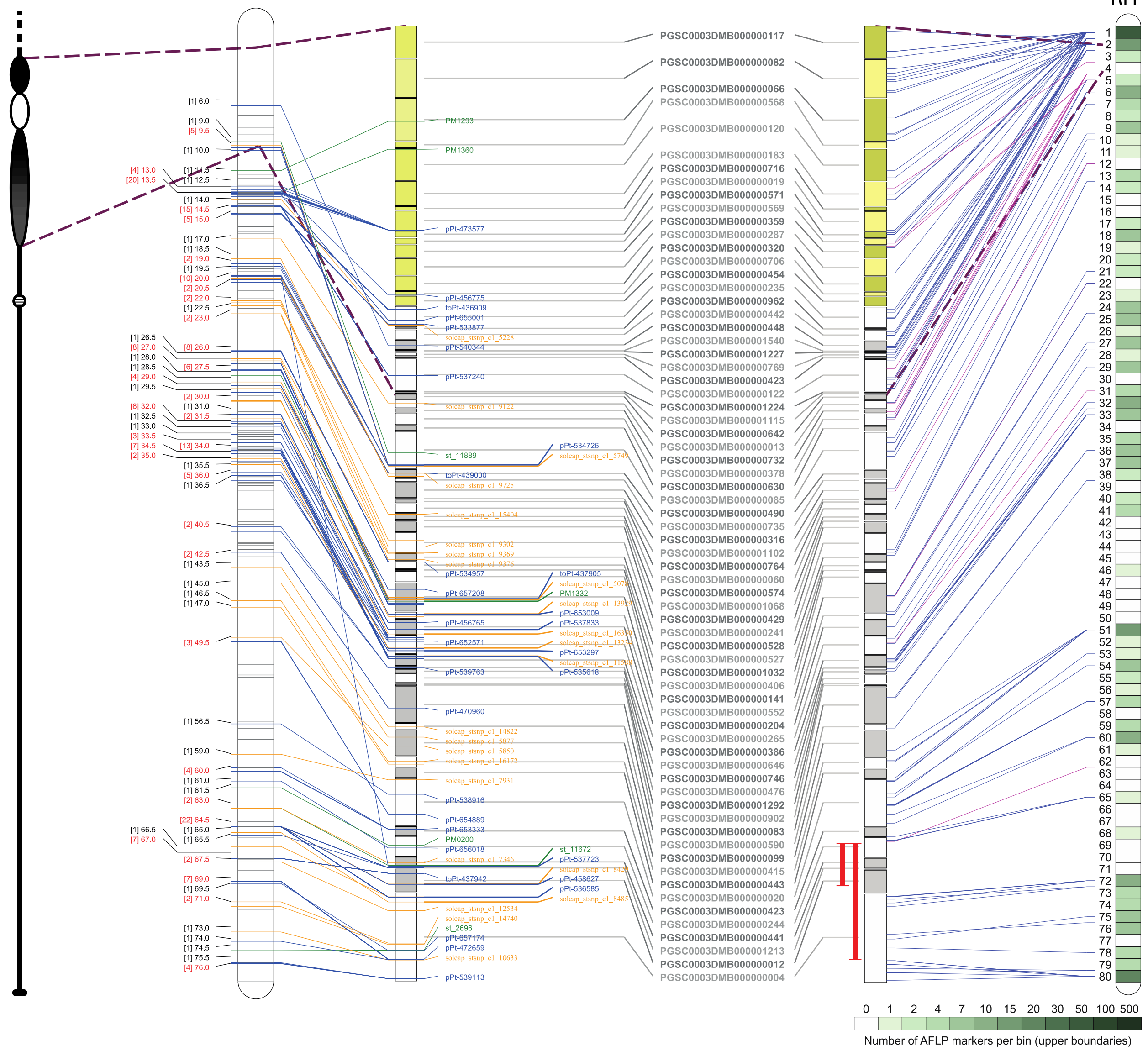

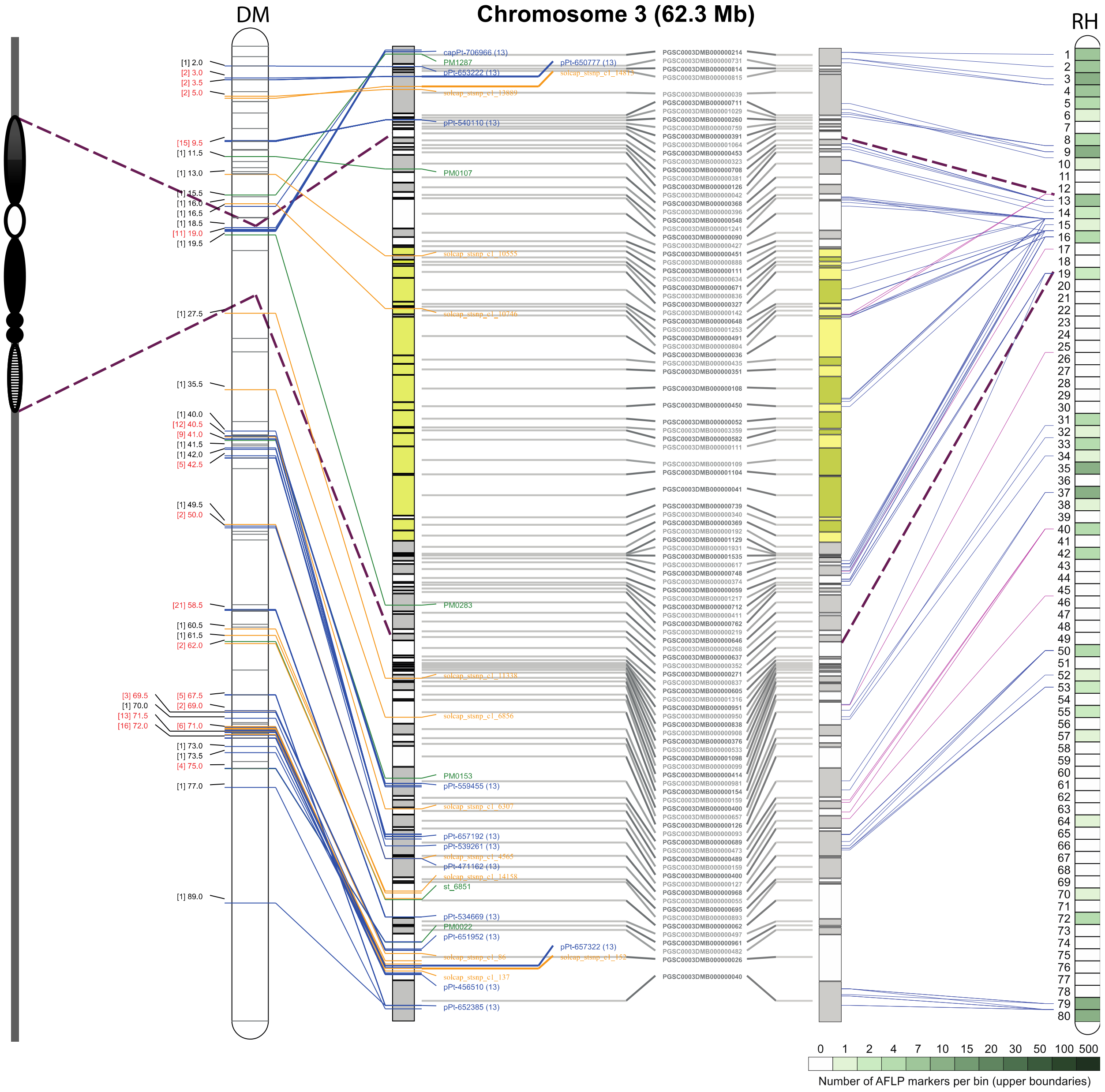
Chromosome 3 (62.3 Mb)
DM
RH
0 1 2 4 7 10 15 20 30 50 100 500
Number of AFLP markers per bin (upper boundaries)

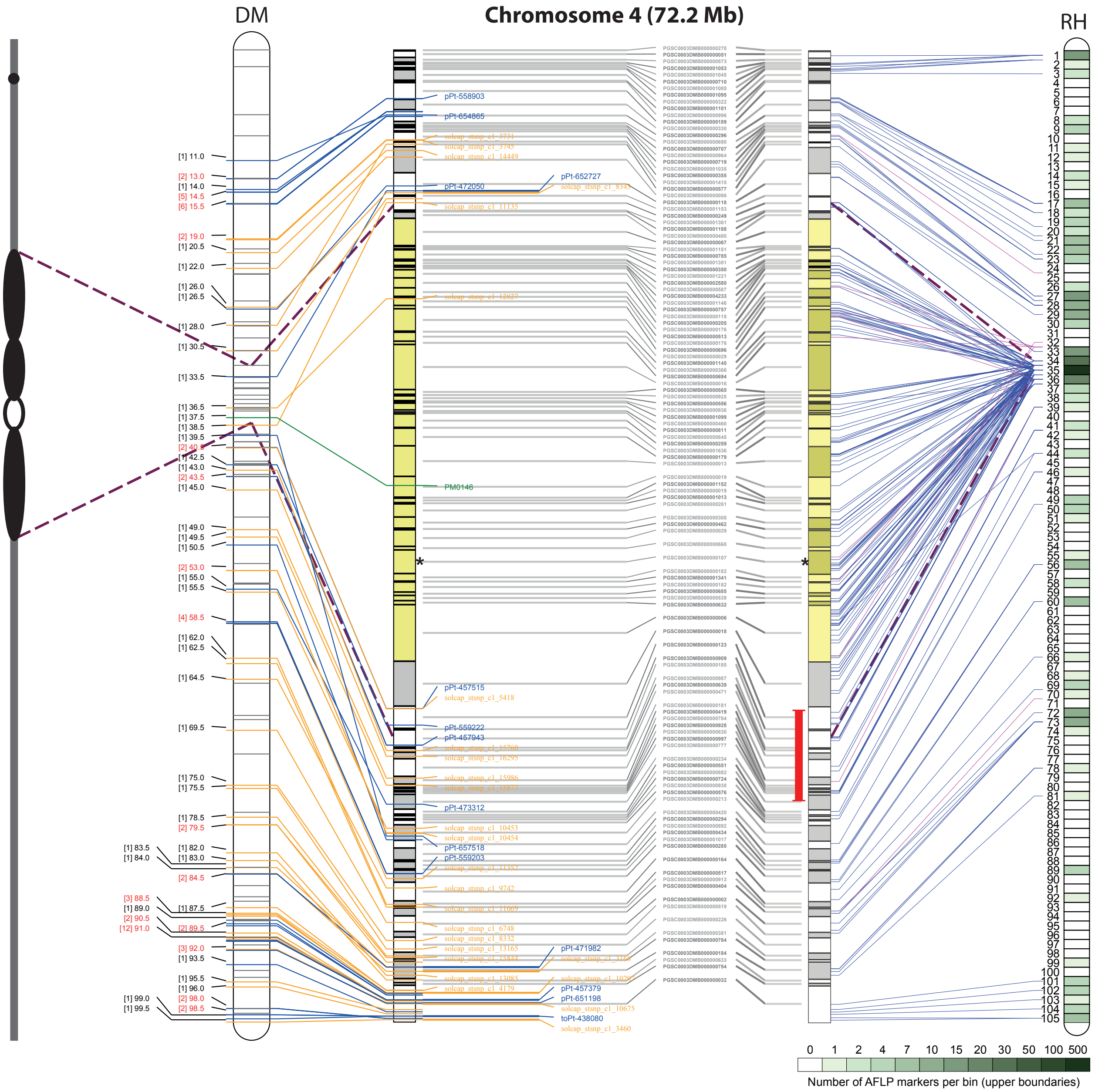
Chromosome 4 (72.2 Mb)
DM
RH
0 1 2 4 7 10 15 20 30 50 100 500
Number of AFLP markers per bin (upper boundaries)

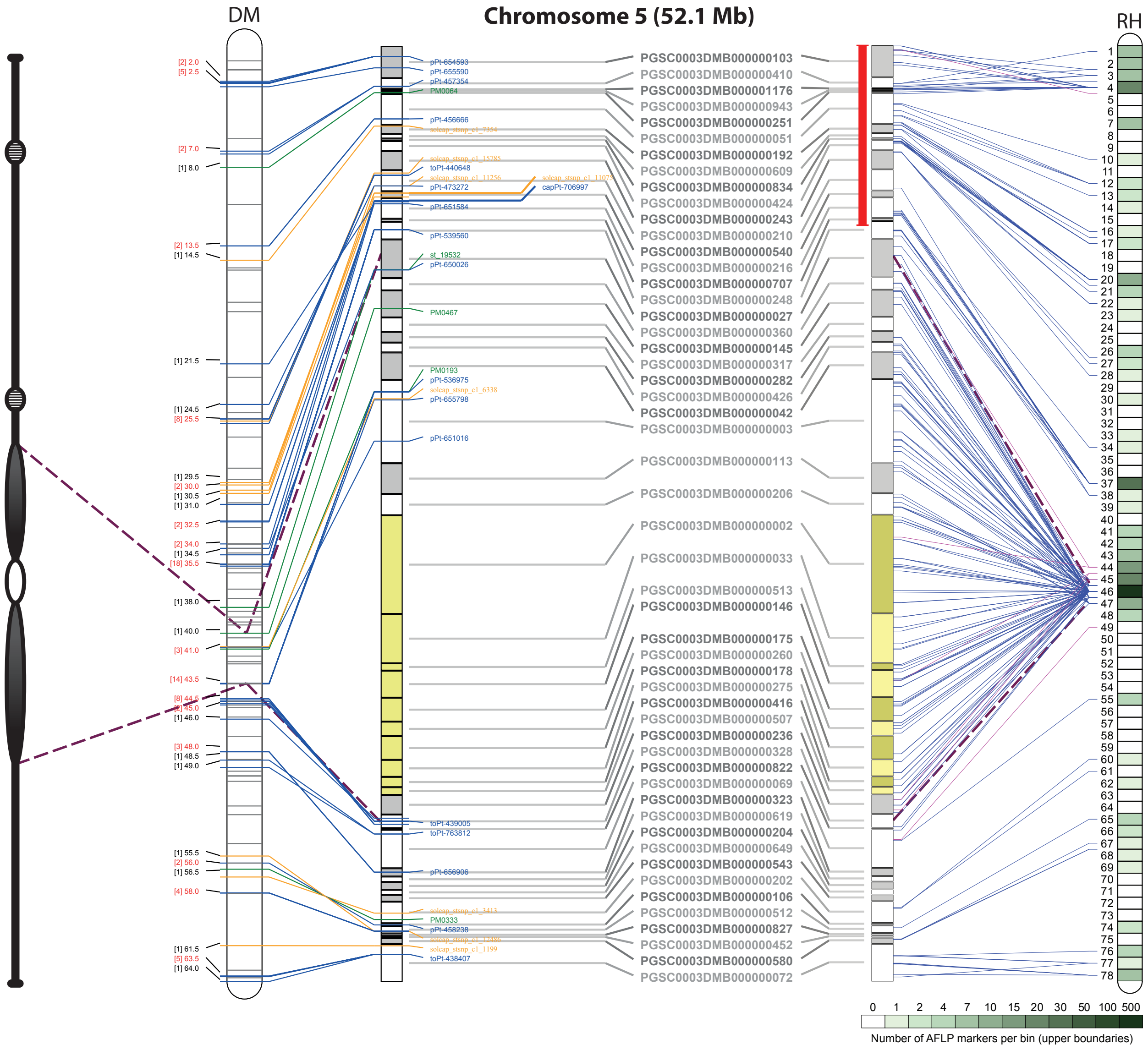

Chromosome 5 (52.1 Mb)
DM
RH
PGSC0003DMB000000103
PGSC0003DMB000000410
PGSC0003DMB000001176
PGSC0003DMB000000943
PGSC0003DMB000000251
PGSC0003DMB000000051
PGSC0003DMB000000192
PGSC0003DMB000000609
PGSC0003DMB000000834
PGSC0003DMB000000424
PGSC0003DMB000000243
PGSC0003DMB000000210
PGSC0003DMB000000540
PGSC0003DMB000000216
PGSC0003DMB000000707
PGSC0003DMB000000248
PGSC0003DMB000000027
PGSC0003DMB000000360
PGSC0003DMB000000145
PGSC0003DMB000000317
PGSC0003DMB000000282
PGSC0003DMB000000426
PGSC0003DMB000000042
PGSC0003DMB000000003
PGSC0003DMB000000113
PGSC0003DMB000000206
PGSC0003DMB000000002
PGSC0003DMB000000033
PGSC0003DMB000000513
PGSC0003DMB000000146
PGSC0003DMB000000175
PGSC0003DMB000000260
PGSC0003DMB000000178
PGSC0003DMB000000275
PGSC0003DMB000000416
PGSC0003DMB000000507
PGSC0003DMB000000236
PGSC0003DMB000000328
PGSC0003DMB000000822
PGSC0003DMB000000069
PGSC0003DMB000000323
PGSC0003DMB000000619
PGSC0003DMB000000204
PGSC0003DMB000000649
PGSC0003DMB000000543
PGSC0003DMB000000202
PGSC0003DMB000000106
PGSC0003DMB000000512
PGSC0003DMB000000827
PGSC0003DMB000000452
PGSC0003DMB000000580
PGSC0003DMB000000072
0 1 2 4 7 10 15 20 30 50 100 500
Number of AFLP markers per bin (upper boundaries)

Chromosome 6 (59.5 Mb)

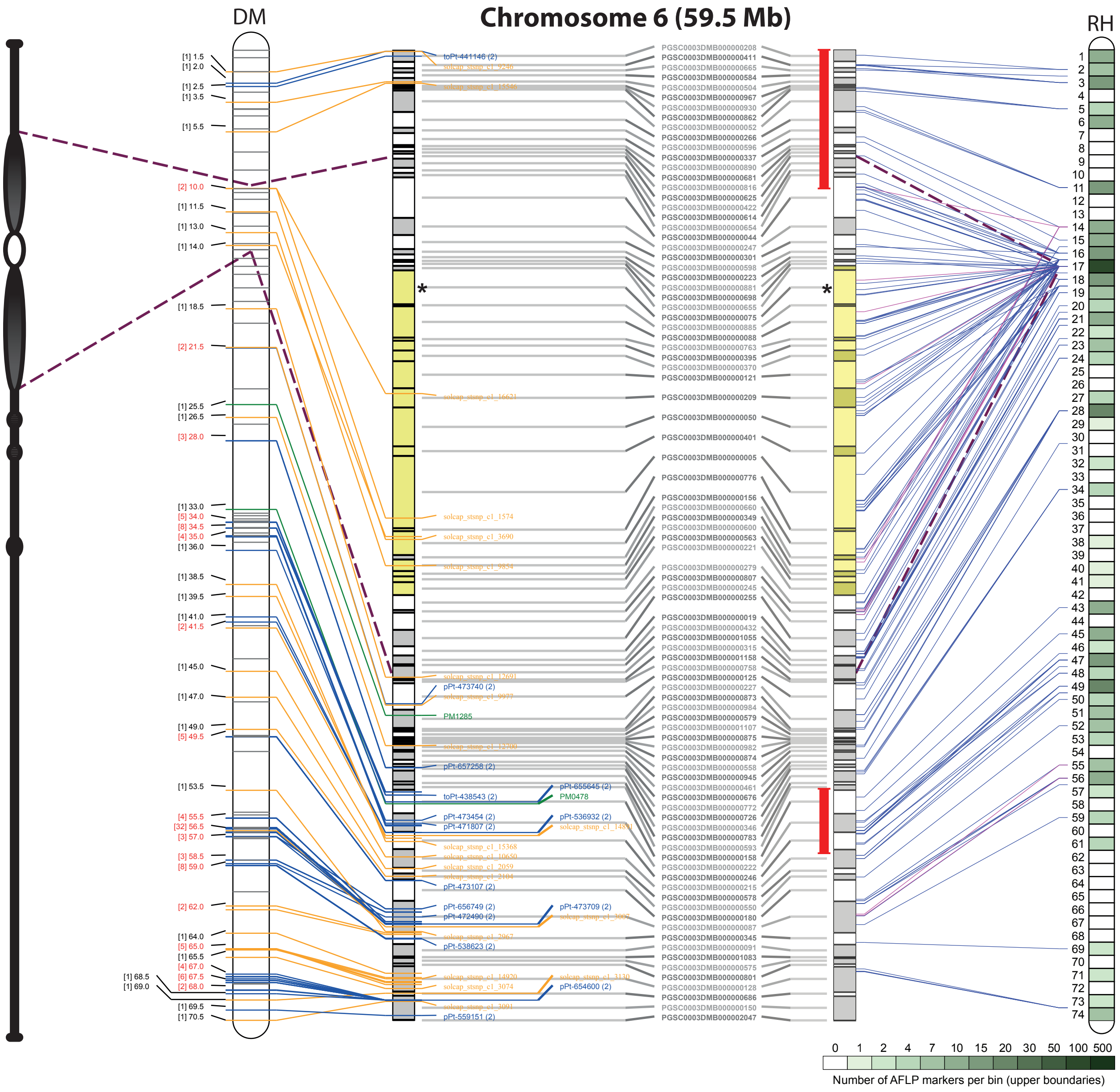

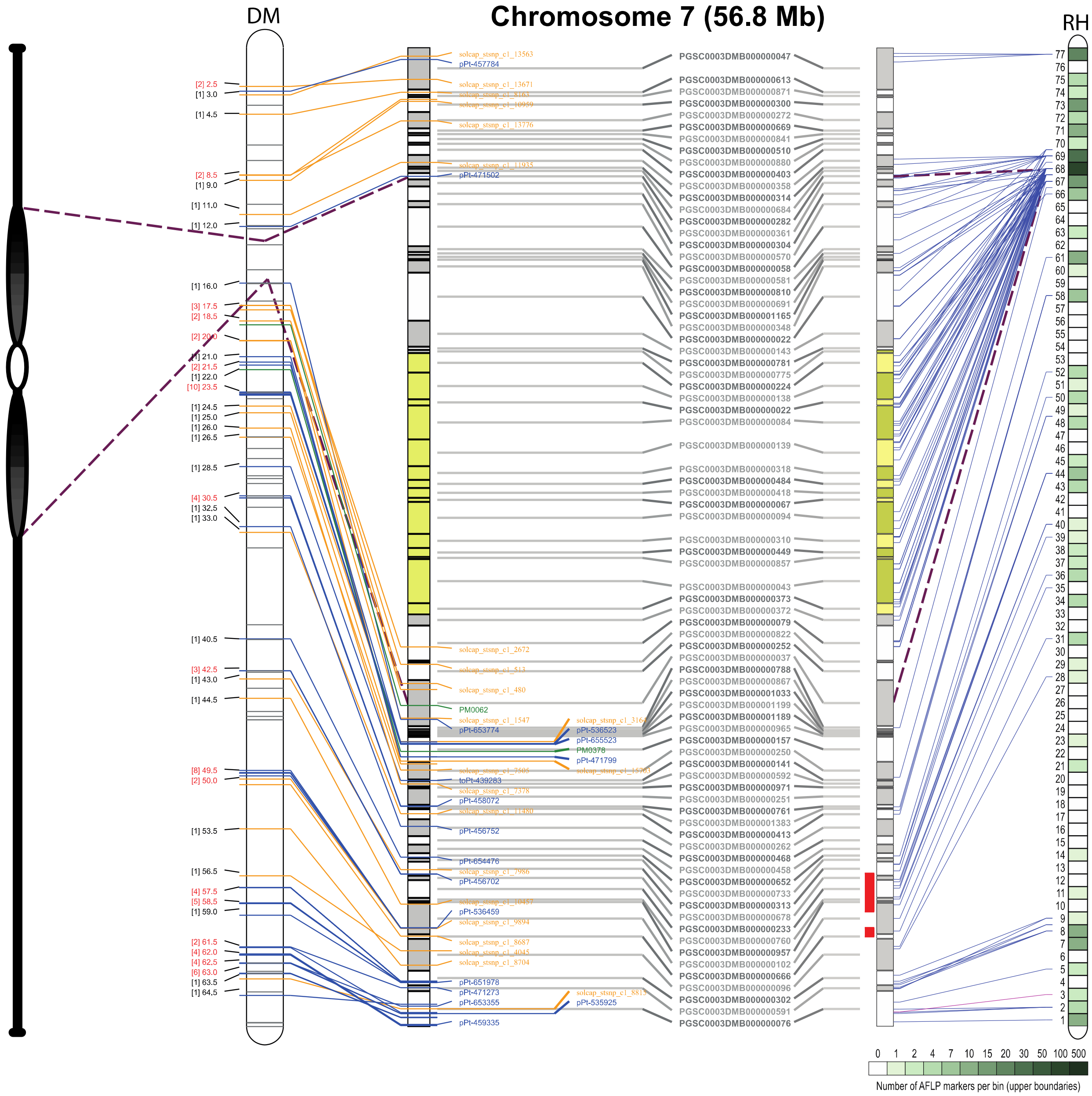

Chromosome 7 (56.8 Mb)
DM
RH
Number of AFLP markers per bin (upper boundaries)

Chromosome 8 (56.9 Mb)

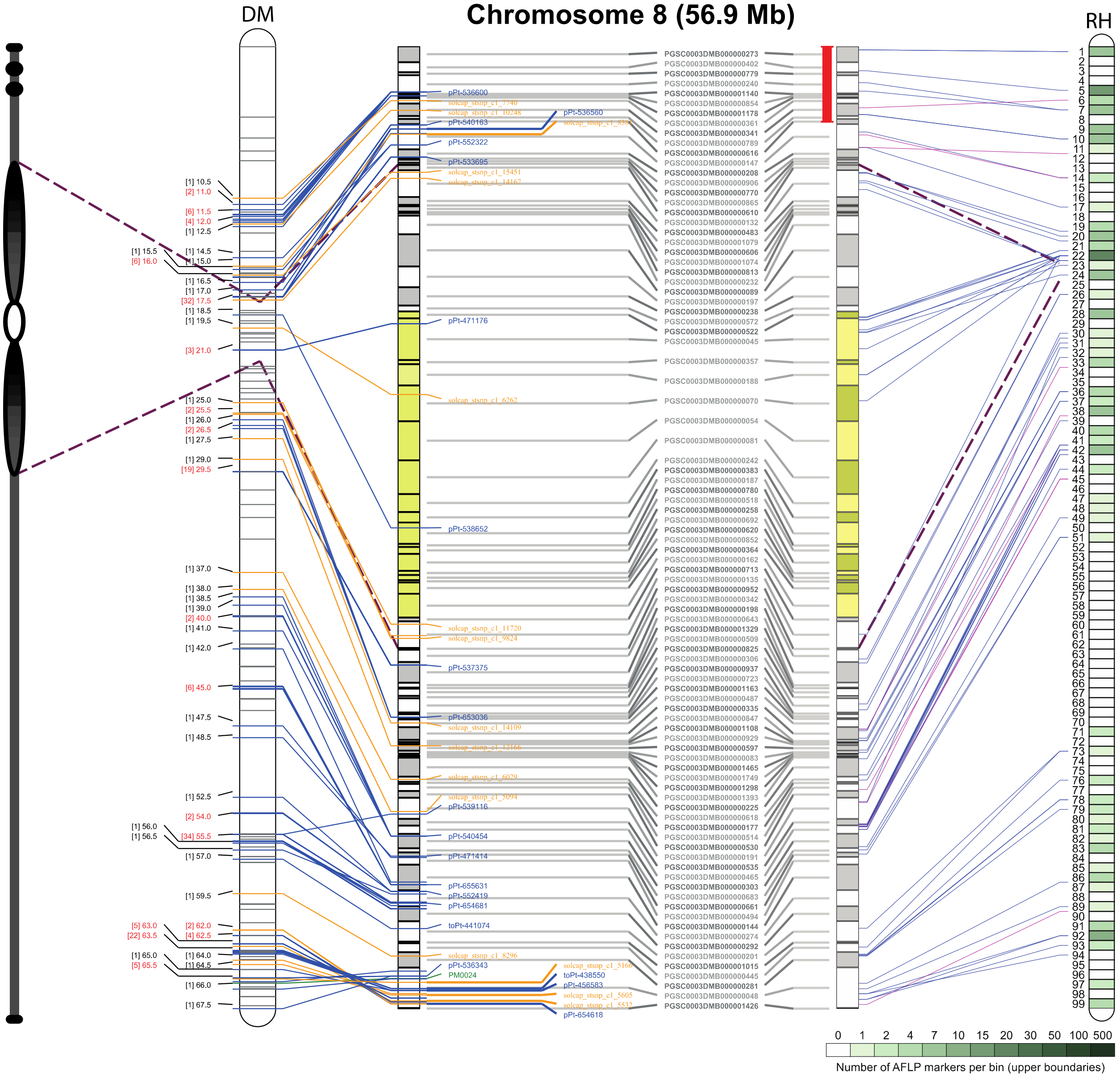

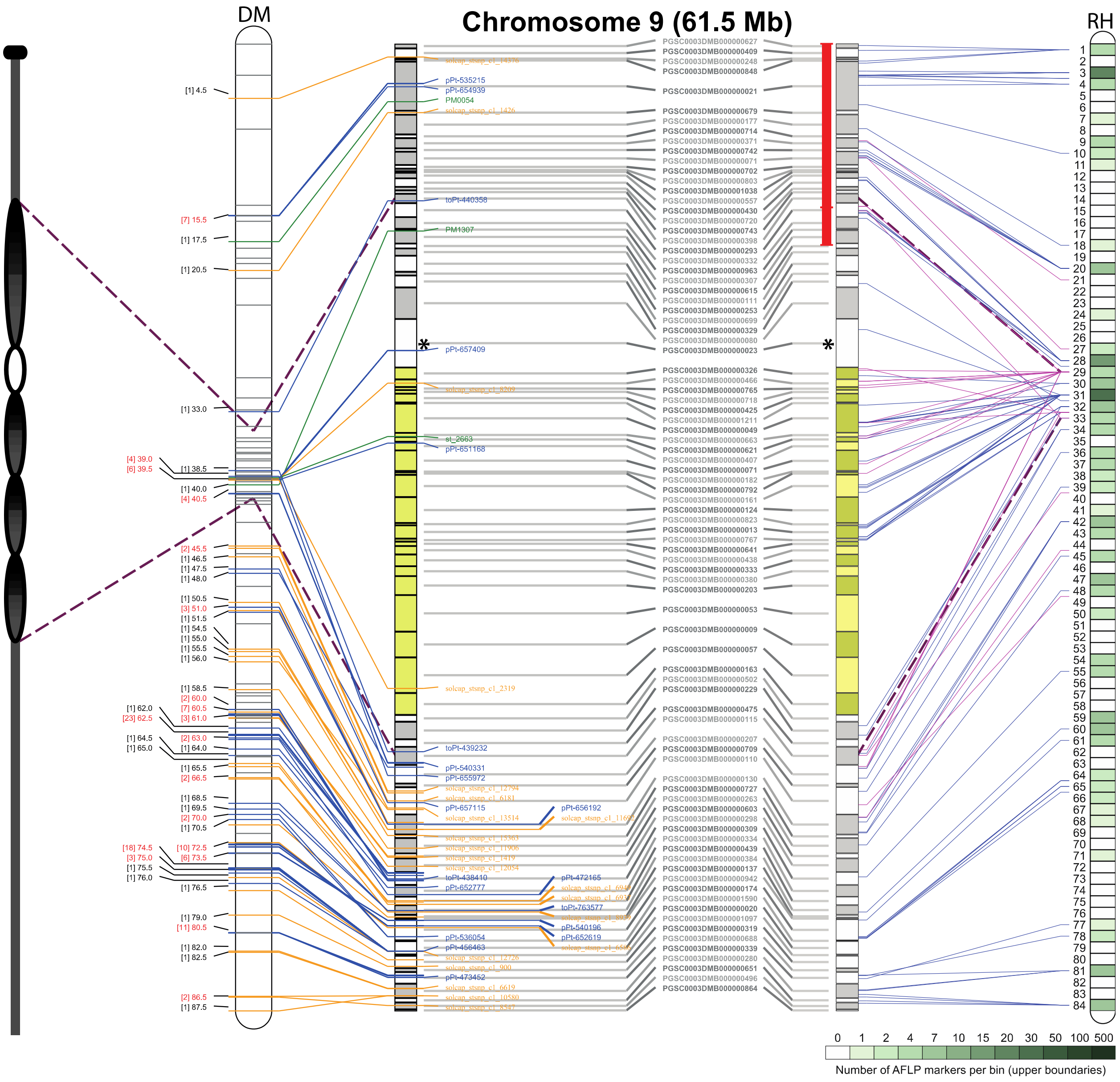
Chromosome 9 (61.5 Mb)
DM
RH
Number of AFLP markers per bin (upper boundaries)

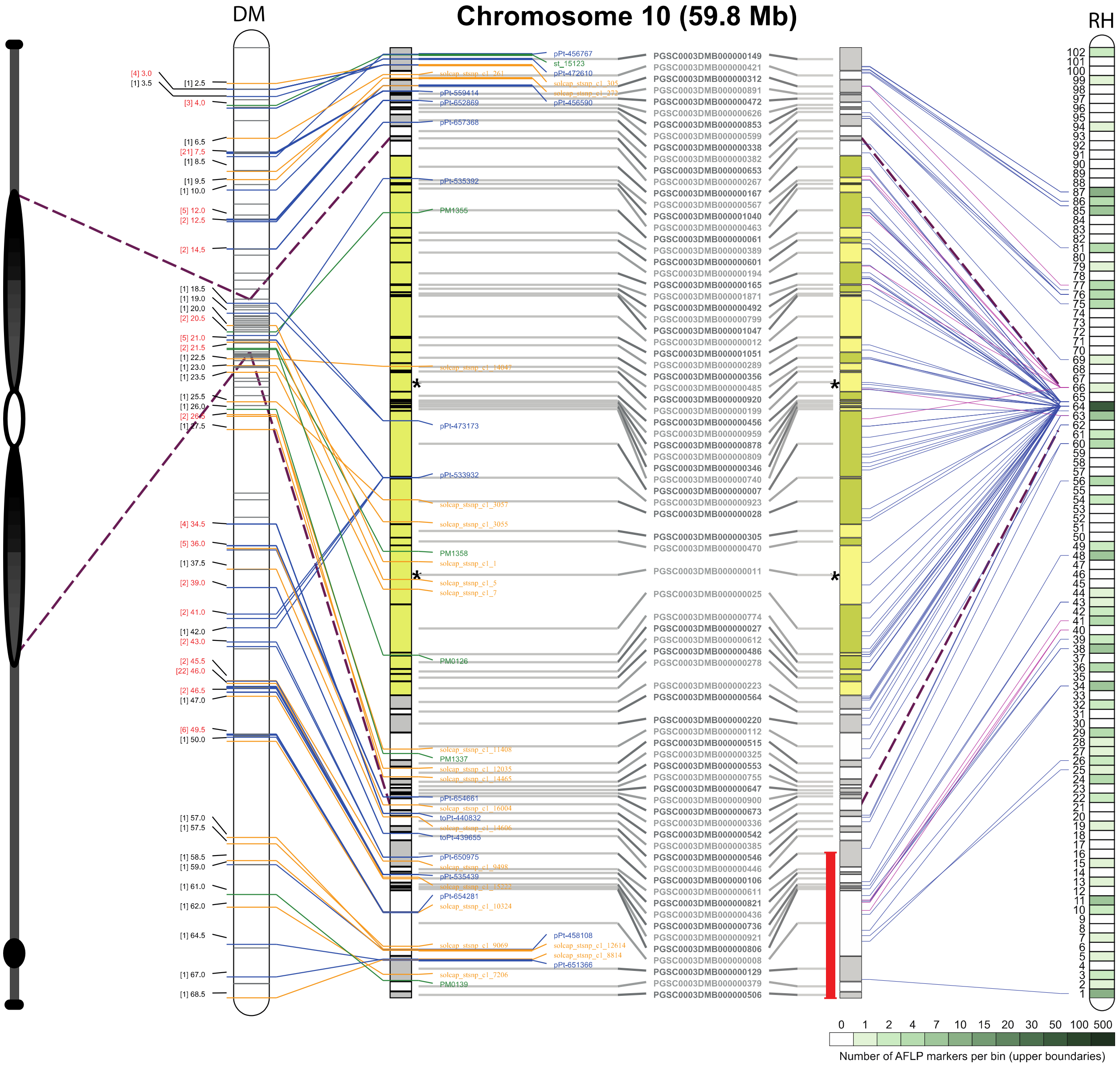
DM
Chromosome 10 (59.8 Mb)
RH
Number of AFLP markers per bin (upper boundaries)

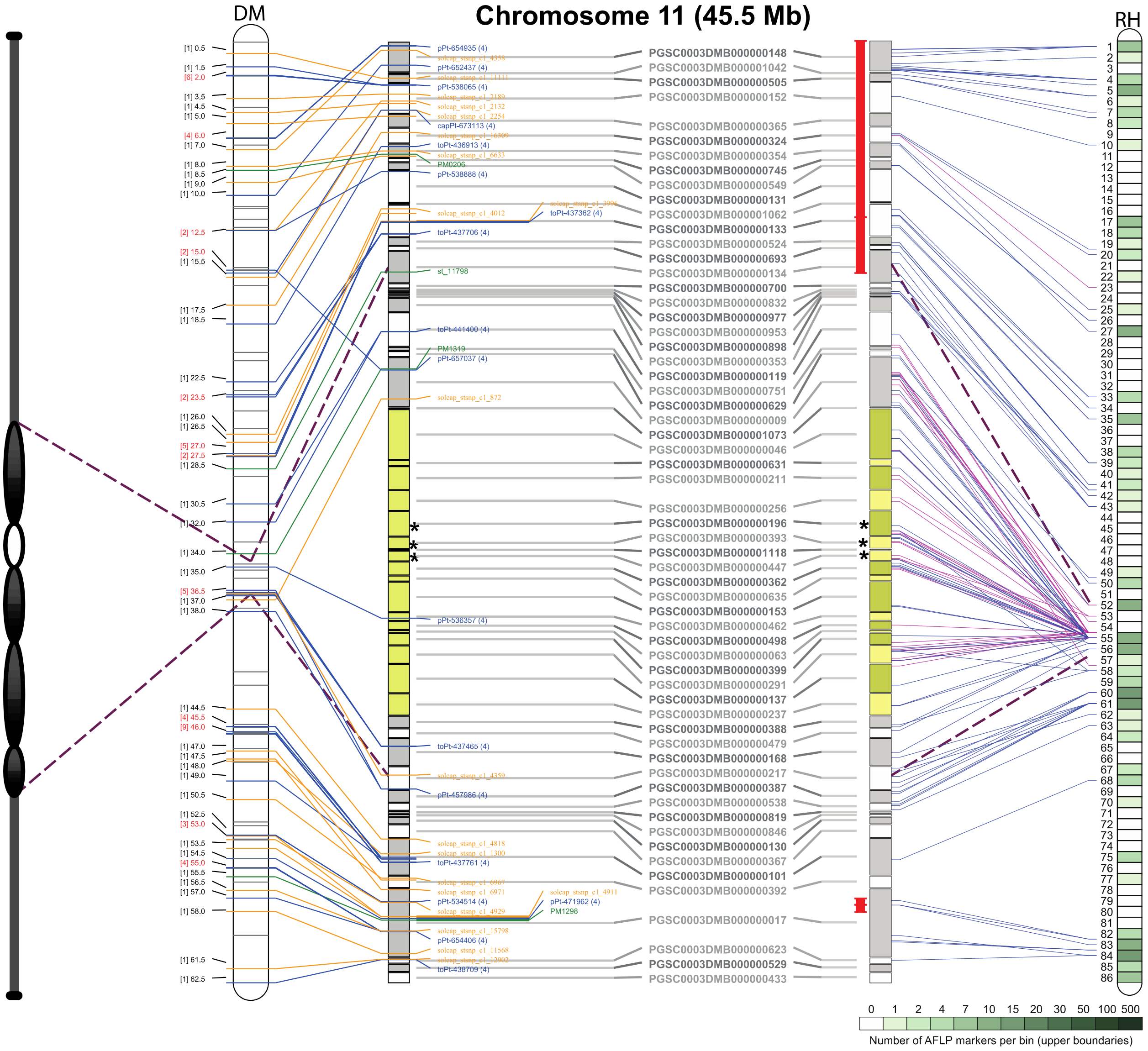
Chromosome 11 (45.5 Mb)
DM
RH
PGSC0003DMB000000148
PGSC0003DMB000001042
PGSC0003DMB000000505
PGSC0003DMB000000152
PGSC0003DMB000000365
PGSC0003DMB000000324
PGSC0003DMB000000354
PGSC0003DMB000000745
PGSC0003DMB000000549
PGSC0003DMB000000131
PGSC0003DMB000001062
PGSC0003DMB000000133
PGSC0003DMB000000524
PGSC0003DMB000000693
PGSC0003DMB000000134
PGSC0003DMB000000700
PGSC0003DMB000000832
PGSC0003DMB000000977
PGSC0003DMB000000953
PGSC0003DMB000000898
PGSC0003DMB000000353
PGSC0003DMB000000119
PGSC0003DMB000000751
PGSC0003DMB000000629
PGSC0003DMB000000009
PGSC0003DMB000001073
PGSC0003DMB000000046
PGSC0003DMB000000631
PGSC0003DMB000000211
PGSC0003DMB000000256
PGSC0003DMB000000196
PGSC0003DMB000000393
PGSC0003DMB000001118
PGSC0003DMB000000447
PGSC0003DMB000000362
PGSC0003DMB000000635
PGSC0003DMB000000153
PGSC0003DMB000000462
PGSC0003DMB000000498
PGSC0003DMB000000063
PGSC0003DMB000000399
PGSC0003DMB000000291
PGSC0003DMB000000137
PGSC0003DMB000000237
PGSC0003DMB000000388
PGSC0003DMB000000479
PGSC0003DMB000000168
PGSC0003DMB000000217
PGSC0003DMB000000387
PGSC0003DMB000000538
PGSC0003DMB000000819
PGSC0003DMB000000846
PGSC0003DMB000000130
PGSC0003DMB000000367
PGSC0003DMB000000101
PGSC0003DMB000000392
PGSC0003DMB000000017
PGSC0003DMB000000623
PGSC0003DMB000000529
PGSC0003DMB000000433
0 1 2 4 7 10 15 20 30 50 100 500
Number of AFLP markers per bin (upper boundaries)

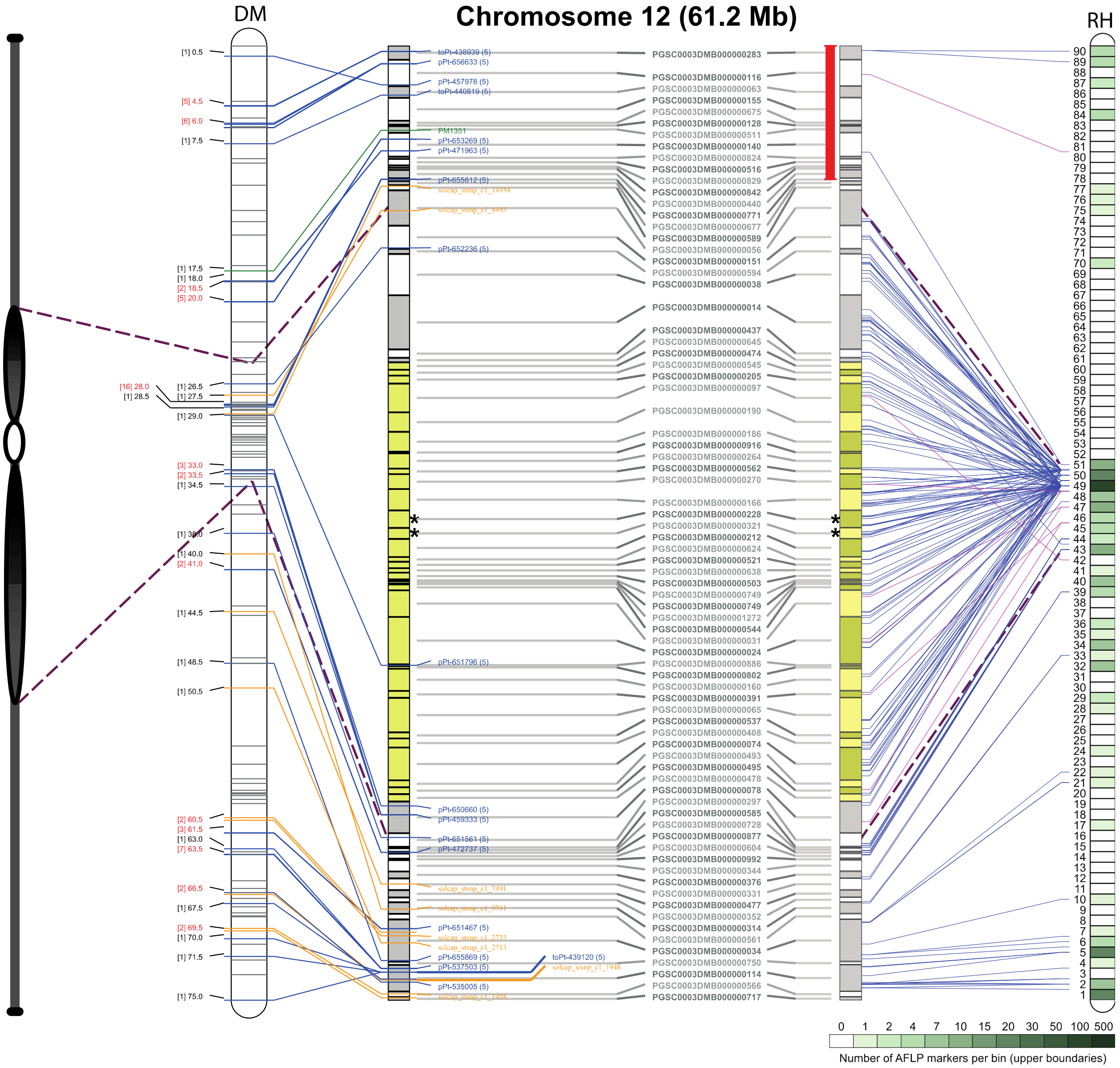

Figure S2 Illustration of the chromosome 2 - 12 pseudomolecules (PMs) integrated with the DM and RH genetic maps. STS and AFLP markers anchor sequence locations in the chromosome PMs to the DMDD and RH genetic maps, respectively. The AFLP marker positions in the PM were identified through sequence tag alignment of BAC clones from the RH WGP physical map. Superscaffolds comprising the PM are shown as alternating grey and white rectangular blocks. The layout of the PM for each of the genetic maps is shown separately but is identical with superscaffold IDs depicted in the middle. The pachytene idiogramis adapted from the potato reference genome publication (Potato Genome Sequencing Consortium 2011). The putative centromere region and pericentromeric/heterochromatic boundaries are demarcated by asterisks and dashed lines, respectively. Each DMDD marker type is colour coded: blue = DArTs, yellow = SNPs, green = SSRs. Blue and magenta lines emerging from the RH genetic map represent AFLP anchors and the intensity of green color corresponds to the AFLP marker density per bin as reported by Van Os *et al*. (2006). Magenta lines represent AFLP markers with a relatively inaccurate mapping position on the RH genetic map, covering an interval of 5 or more bins. Regions in the central heterochromatin where superscaffold order and orientation are not completely resolved are indicated in yellow. Inversions with the tomato sequence are indicated with red interval bars.

Tables S1-S9
Available for download at http://www.g3journal.org/lookup/suppl/doi:10.1534/g3.113.007153/-/DC1

Table S1 Details of (A) Simple sequence repeat (SSR), (B) Single nucleotide polymorphism (SNP) and (C) Amplified fragment length polymorphism (AFLP) markers employed in DMDD genotyping.

Table S2 Location of sequence-tagged site (STS) markers employed in DMDD genotyping on the DM version 3 superscaffolds and DM version 4.03 pseudomolecules. STS markers include DArTs, SSRs and SNPs.

Table S3 Revised annotation details for the Infinium 8.3k Potato Array SNPs (Felcher *et al*. 2012) on DM version 4.03 pseudomolecules.

Table S4 Genetic and physical locations of STS markers (DArTs, SSRs and SNPs) mapped in DMDD and anchored in DM version 4.03 pseudomolecules.

Table S5 Paracentric inversions between potato and tomato chromosomes detected by dot plot alignments between the chromosome pseudomolecules V4.03 of potato line DM and V2.40 of tomato cv. 'Heinz 1706'.

Table S6 Summary of six BAC pools sequence assembly data comprising 82 DM BAC clones used for validating link peak-based orientation strategy for chromosome 4.

Table S7 BAC pool assembly and validation details for chromosome 4 pseudomolecule version 4.03.

Table S8 Centromere localisation in DM V3 sequence assembly.

Table S9 Accessioned Golden Path (AGP) for the reference DM chromosome-scale pseudomolecules version 4.03. File also includes revised annotation details for potato genes and repeat regions (Potato Genome Sequencing Consortium 2011) and a list of chimeric superscaffolds.
