# Supplementary material for: Construction of Reference Chromosome-Scale Pseudomolecules for Potato: Integrating the Potato Genome with Genetic and Physical Maps
Source: G3 (Bethesda). 2013 Nov 1;3(11):2031–47. doi: 10.1534/g3.113.007153 (PMC3815063; doi:10.1534/g3.113.007153)
Supplement: Supporting Information [file supp_g3.113.007153_FigureS1.pdf]

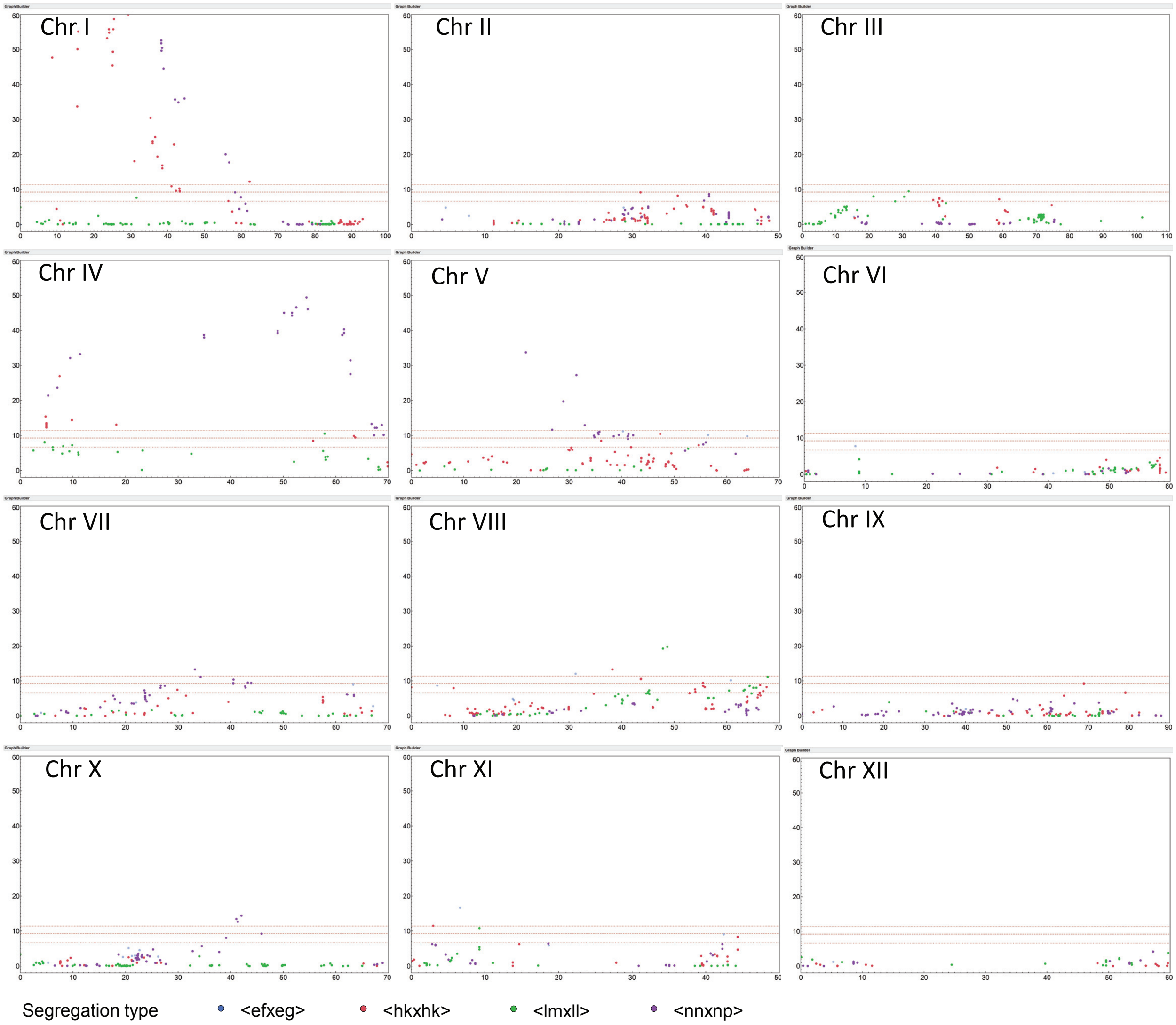

Figure S1 Genome-wide patterns of marker segregation distortion in DMDD population for 1830 STS markers from different segregation categories plotted as a function of Chi-square value (y-axis) against marker physical position (x-axis) on each of the 12 potato chromosomes. Dotted, dashed and dotted-dashed lines represent Chi-square significance values at p = 0.01 for marker segregation categories with two, three and four genotypic classes, respectively.
