# Supplementary material for: Construction of Reference Chromosome-Scale Pseudomolecules for Potato: Integrating the Potato Genome with Genetic and Physical Maps
Source: G3 (Bethesda). 2013 Nov 1;3(11):2031–47. doi: 10.1534/g3.113.007153 (PMC3815063; doi:10.1534/g3.113.007153)
Supplement: Supporting Information [file supp_g3.113.007153_FigureS2.pdf]

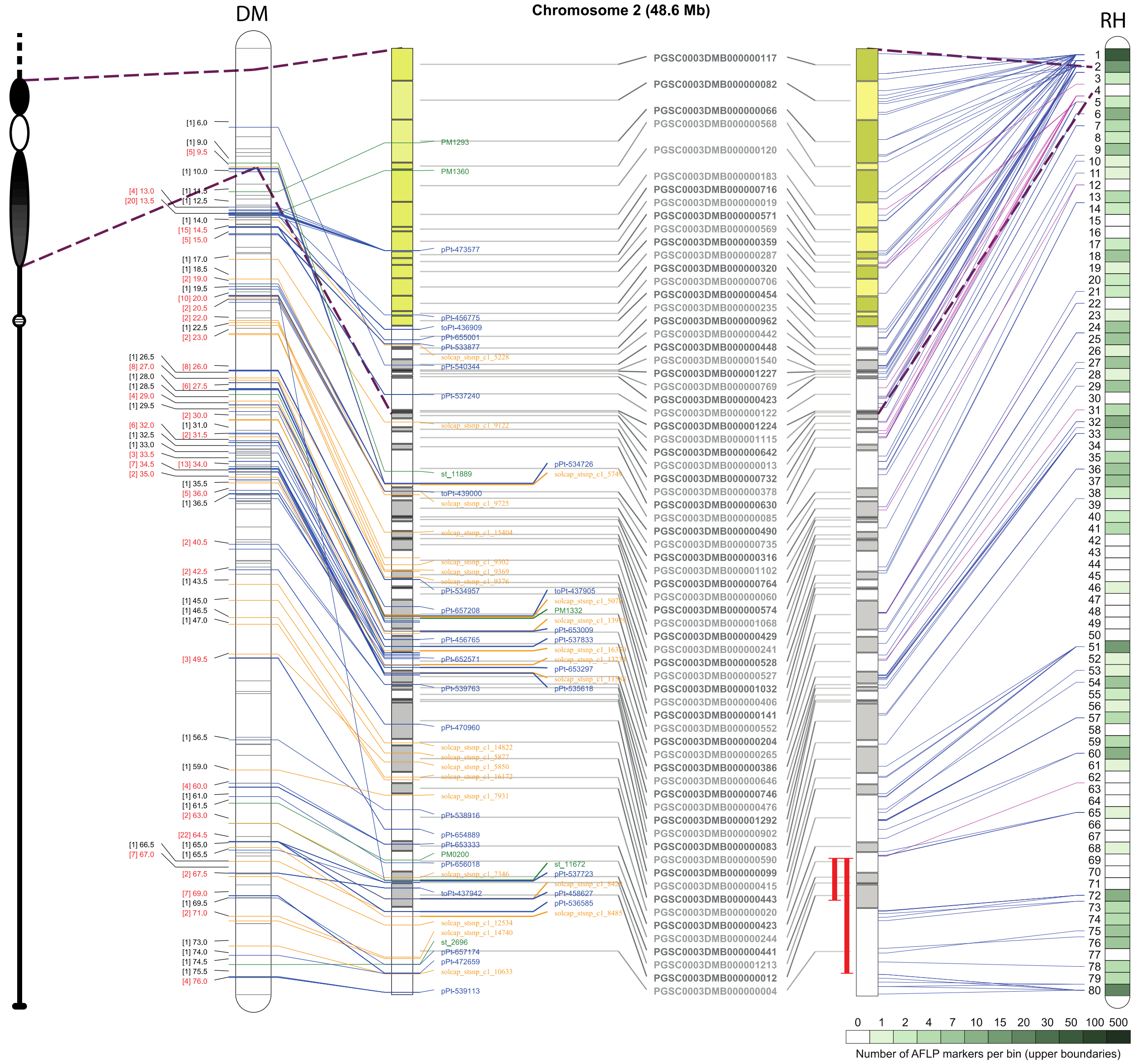

Chromosome 2 (48.6 Mb)
DM
RH
PGSC0003DMB000000117
PGSC0003DMB000000082
PGSC0003DMB000000066
PGSC0003DMB000000568
PGSC0003DMB000000120
PGSC0003DMB000000183
PGSC0003DMB000000716
PGSC0003DMB000000019
PGSC0003DMB000000571
PGSC0003DMB000000569
PGSC0003DMB000000359
PGSC0003DMB000000287
PGSC0003DMB000000320
PGSC0003DMB000000706
PGSC0003DMB000000454
PGSC0003DMB000000235
PGSC0003DMB000000962
PGSC0003DMB000000442
PGSC0003DMB000000448
PGSC0003DMB000001540
PGSC0003DMB000001227
PGSC0003DMB000000769
PGSC0003DMB000000423
PGSC0003DMB000000122
PGSC0003DMB000001224
PGSC0003DMB000001115
PGSC0003DMB000000642
PGSC0003DMB000000013
PGSC0003DMB000000732
PGSC0003DMB000000378
PGSC0003DMB000000630
PGSC0003DMB000000085
PGSC0003DMB000000490
PGSC0003DMB000000735
PGSC0003DMB000000316
PGSC0003DMB000001102
PGSC0003DMB000000764
PGSC0003DMB000000060
PGSC0003DMB000000574
PGSC0003DMB000001068
PGSC0003DMB000000429
PGSC0003DMB000000241
PGSC0003DMB000000528
PGSC0003DMB000000527
PGSC0003DMB000001032
PGSC0003DMB000000406
PGSC0003DMB000000141
PGSC0003DMB000000552
PGSC0003DMB000000204
PGSC0003DMB000000265
PGSC0003DMB000000386
PGSC0003DMB000000646
PGSC0003DMB000000746
PGSC0003DMB000000476
PGSC0003DMB000001292
PGSC0003DMB000000902
PGSC0003DMB000000083
PGSC0003DMB000000590
PGSC0003DMB000000099
PGSC0003DMB000000415
PGSC0003DMB000000443
PGSC0003DMB000000020
PGSC0003DMB000000423
PGSC0003DMB000000244
PGSC0003DMB000000441
PGSC0003DMB000001213
PGSC0003DMB000000012
PGSC0003DMB000000004
0 1 2 4 7 10 15 20 30 50 100 500
Number of AFLP markers per bin (upper boundaries)

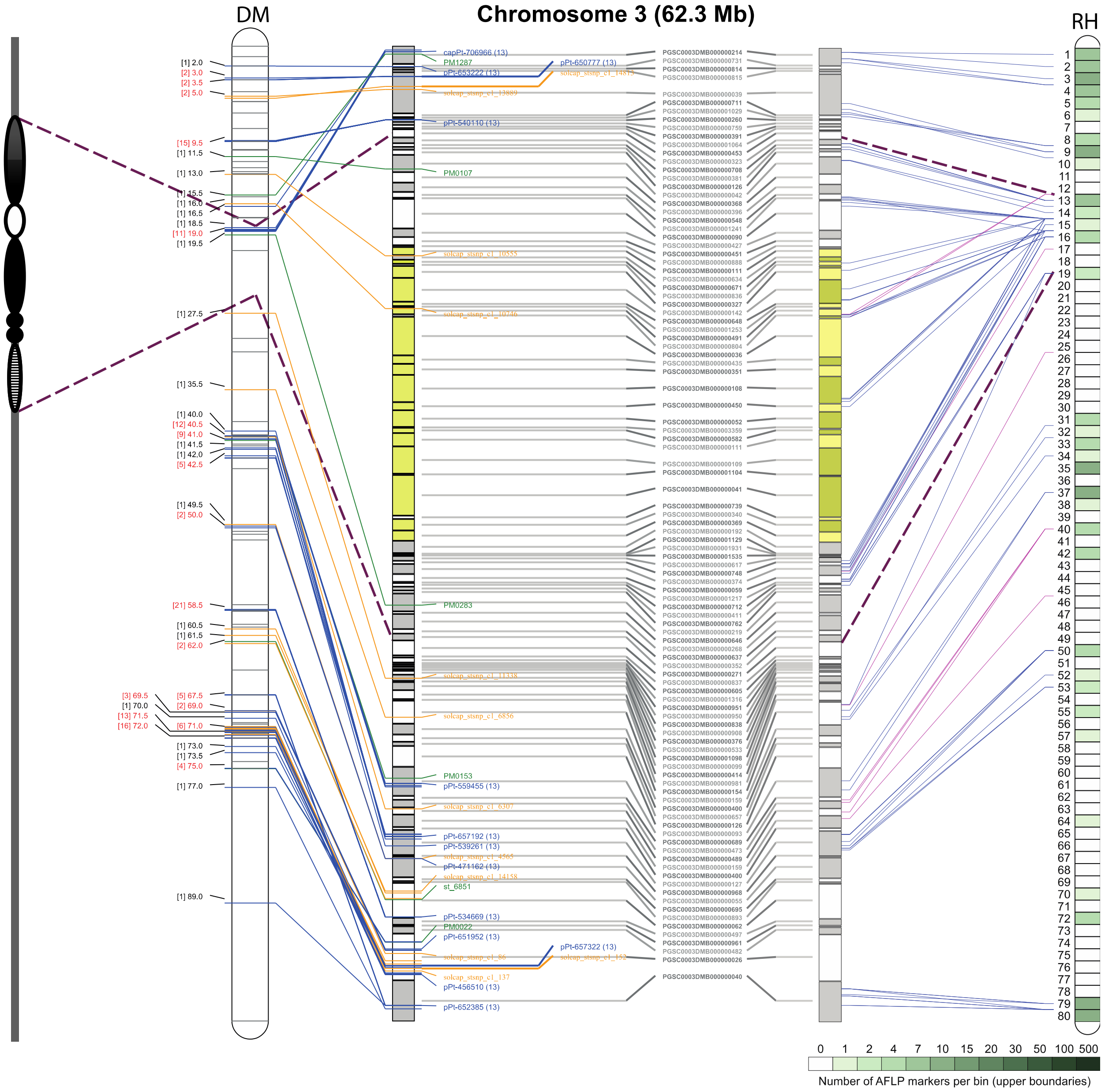
Chromosome 3 (62.3 Mb)
DM
RH
Number of AFLP markers per bin (upper boundaries)

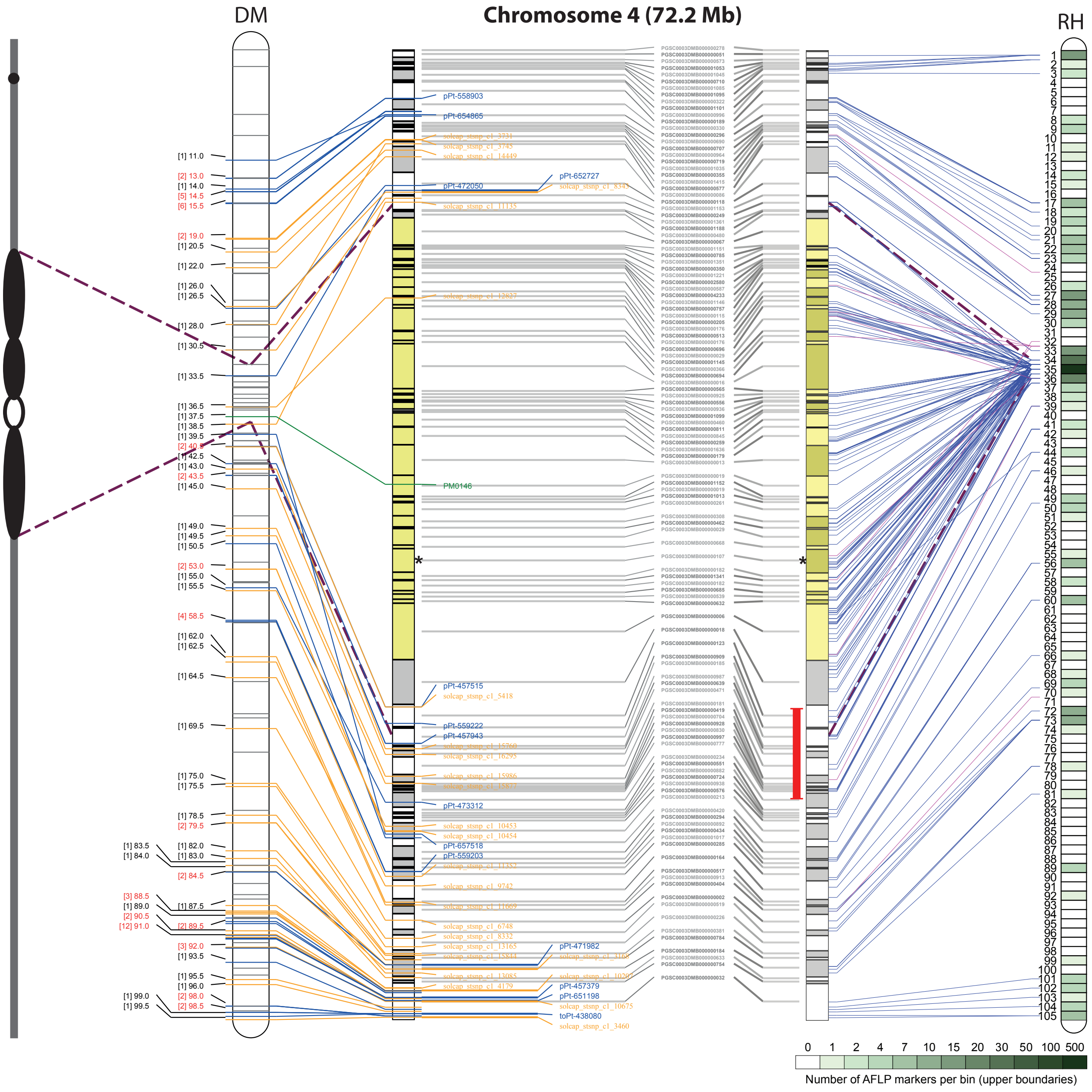
DM
Chromosome 4 (72.2 Mb)
RH
Number of AFLP markers per bin (upper boundaries)
0
1
2
4
7
10
15
20
30
50
100
500

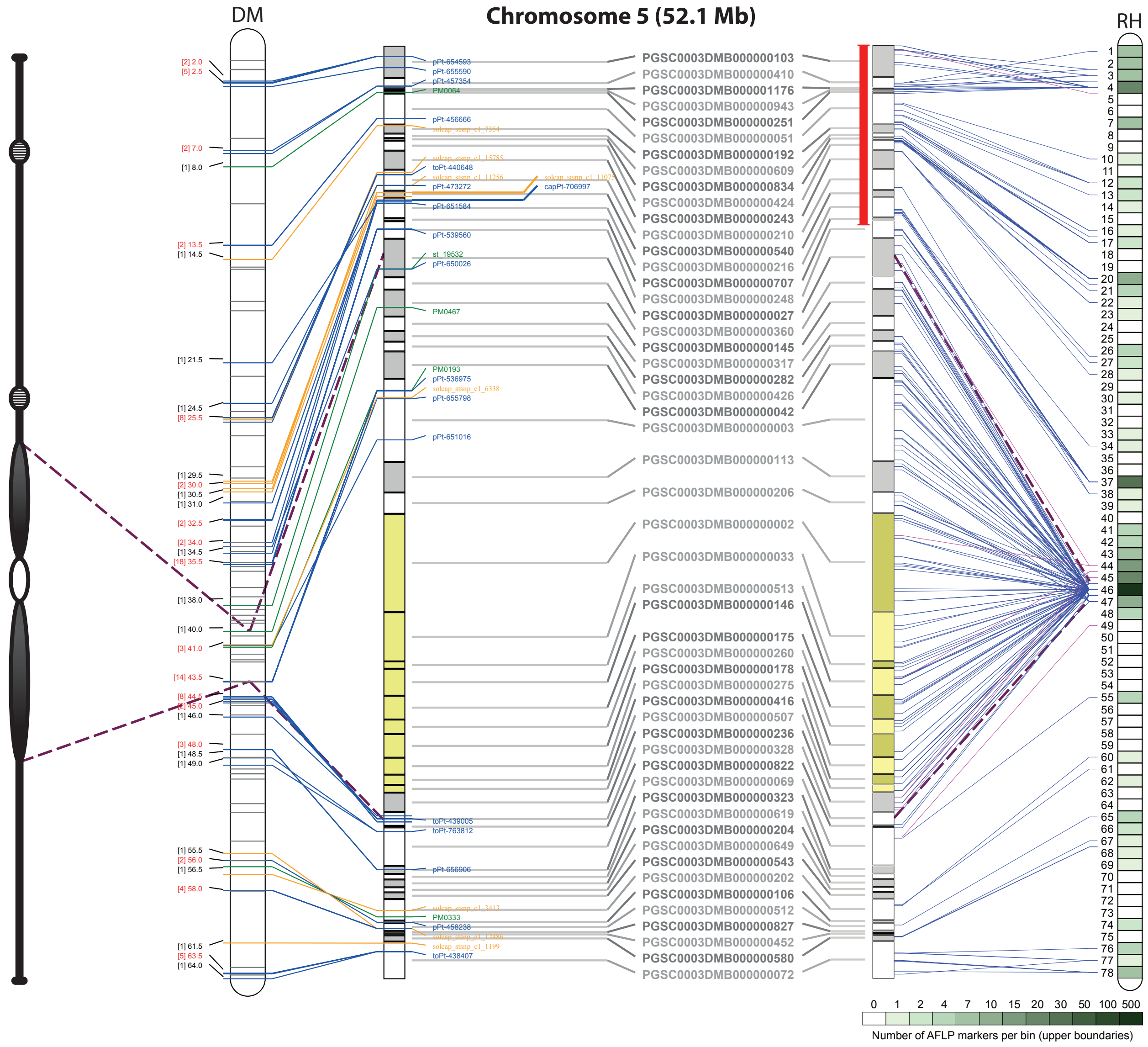
Chromosome 5 (52.1 Mb)
DM
RH
PGSC0003DMB000000103
PGSC0003DMB000000410
PGSC0003DMB000001176
PGSC0003DMB000000943
PGSC0003DMB000000251
PGSC0003DMB000000051
PGSC0003DMB000000192
PGSC0003DMB000000609
PGSC0003DMB000000834
PGSC0003DMB000000424
PGSC0003DMB000000243
PGSC0003DMB000000210
PGSC0003DMB000000540
PGSC0003DMB000000216
PGSC0003DMB000000707
PGSC0003DMB000000248
PGSC0003DMB000000027
PGSC0003DMB000000360
PGSC0003DMB000000145
PGSC0003DMB000000317
PGSC0003DMB000000282
PGSC0003DMB000000426
PGSC0003DMB000000042
PGSC0003DMB000000003
PGSC0003DMB000000113
PGSC0003DMB000000206
PGSC0003DMB000000002
PGSC0003DMB000000033
PGSC0003DMB000000513
PGSC0003DMB000000146
PGSC0003DMB000000175
PGSC0003DMB000000260
PGSC0003DMB000000178
PGSC0003DMB000000275
PGSC0003DMB000000416
PGSC0003DMB000000507
PGSC0003DMB000000236
PGSC0003DMB000000328
PGSC0003DMB000000822
PGSC0003DMB000000069
PGSC0003DMB000000323
PGSC0003DMB000000619
PGSC0003DMB000000204
PGSC0003DMB000000649
PGSC0003DMB000000543
PGSC0003DMB000000202
PGSC0003DMB000000106
PGSC0003DMB000000512
PGSC0003DMB000000827
PGSC0003DMB000000452
PGSC0003DMB000000580
PGSC0003DMB000000072
0 1 2 4 7 10 15 20 30 50 100 500
Number of AFLP markers per bin (upper boundaries)

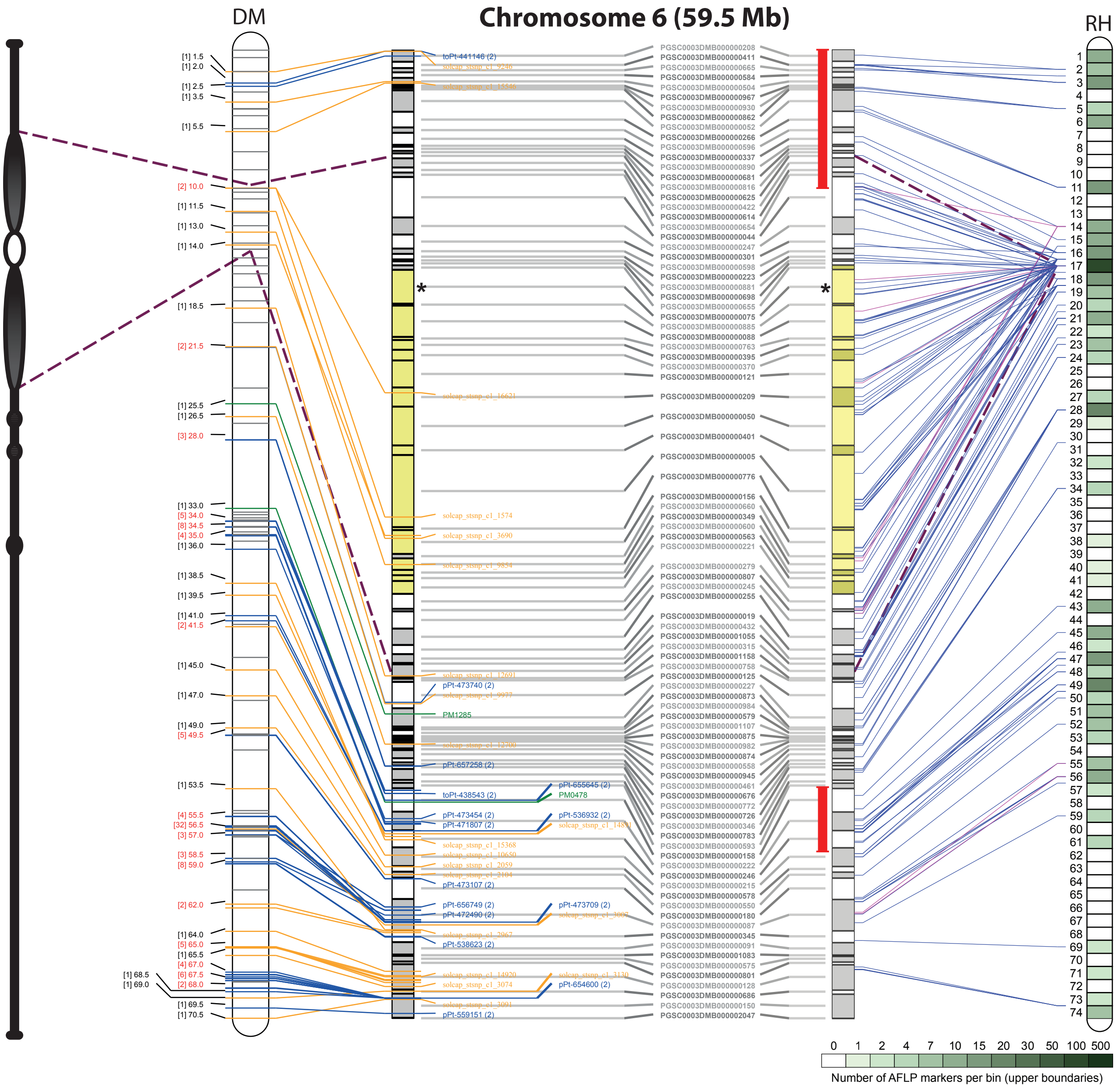

Chromosome 6 (59.5 Mb)
DM
RH
Number of AFLP markers per bin (upper boundaries)

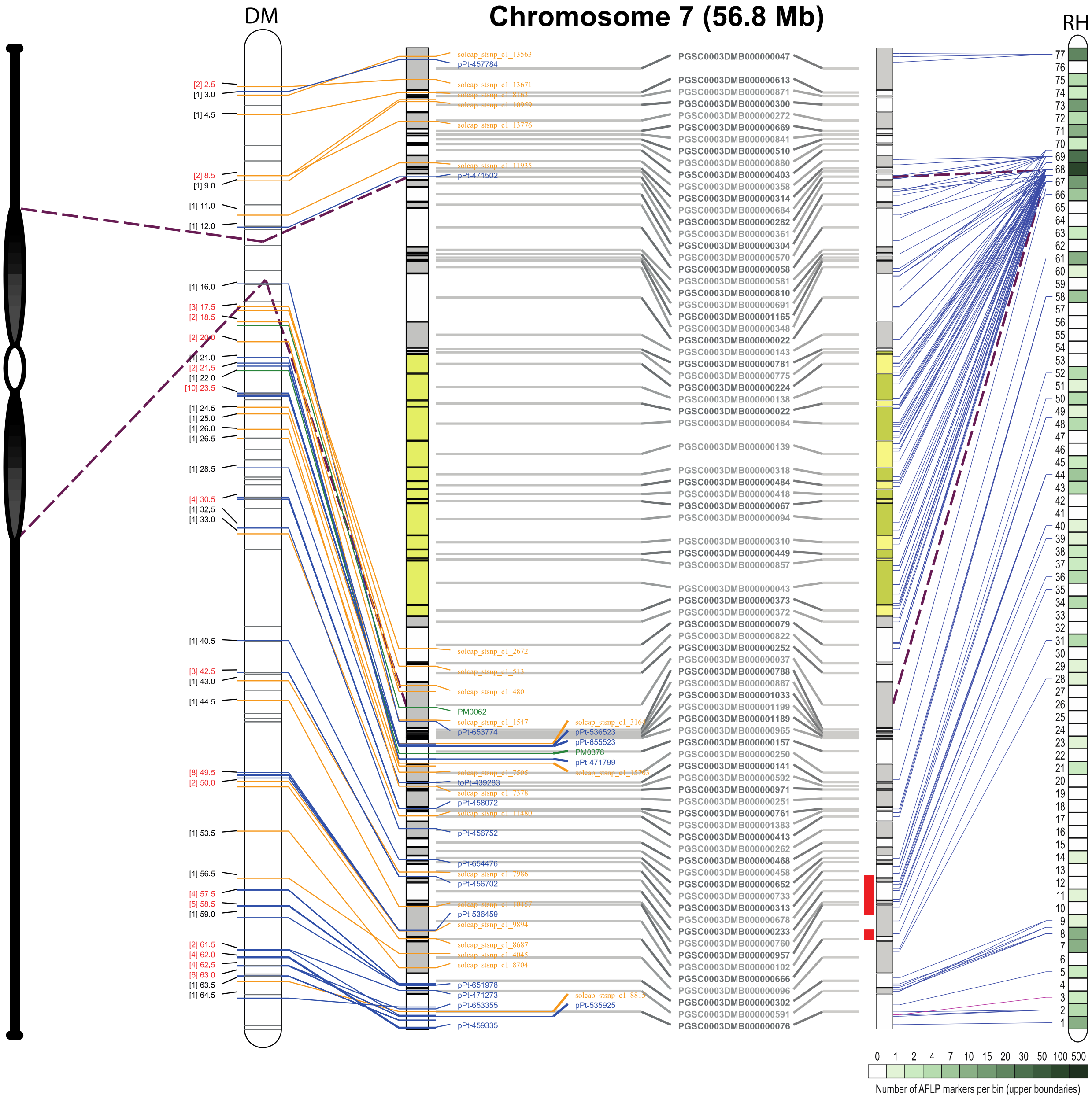
Chromosome 7 (56.8 Mb)
DM
RH
PGSC0003DMB000000047
PGSC0003DMB000000613
PGSC0003DMB000000871
PGSC0003DMB000000300
PGSC0003DMB000000272
PGSC0003DMB000000669
PGSC0003DMB000000841
PGSC0003DMB000000510
PGSC0003DMB000000880
PGSC0003DMB000000403
PGSC0003DMB000000358
PGSC0003DMB000000314
PGSC0003DMB000000684
PGSC0003DMB000000282
PGSC0003DMB000000361
PGSC0003DMB000000304
PGSC0003DMB000000570
PGSC0003DMB000000058
PGSC0003DMB000000581
PGSC0003DMB000000810
PGSC0003DMB000000691
PGSC0003DMB000001165
PGSC0003DMB000000348
PGSC0003DMB000000022
PGSC0003DMB000000143
PGSC0003DMB000000781
PGSC0003DMB000000775
PGSC0003DMB000000224
PGSC0003DMB000000138
PGSC0003DMB000000022
PGSC0003DMB000000084
PGSC0003DMB000000139
PGSC0003DMB000000318
PGSC0003DMB000000484
PGSC0003DMB000000418
PGSC0003DMB000000067
PGSC0003DMB000000094
PGSC0003DMB000000310
PGSC0003DMB000000449
PGSC0003DMB000000857
PGSC0003DMB000000043
PGSC0003DMB000000373
PGSC0003DMB000000372
PGSC0003DMB000000079
PGSC0003DMB000000822
PGSC0003DMB000000252
PGSC0003DMB000000037
PGSC0003DMB000000788
PGSC0003DMB000000867
PGSC0003DMB000001033
PGSC0003DMB000001199
PGSC0003DMB000001189
PGSC0003DMB000000965
PGSC0003DMB000000157
PGSC0003DMB000000250
PGSC0003DMB000000141
PGSC0003DMB000000592
PGSC0003DMB000000971
PGSC0003DMB000000251
PGSC0003DMB000000761
PGSC0003DMB000001383
PGSC0003DMB000000413
PGSC0003DMB000000262
PGSC0003DMB000000468
PGSC0003DMB000000458
PGSC0003DMB000000652
PGSC0003DMB000000733
PGSC0003DMB000000313
PGSC0003DMB000000678
PGSC0003DMB000000233
PGSC0003DMB000000760
PGSC0003DMB000000957
PGSC0003DMB000000102
PGSC0003DMB000000666
PGSC0003DMB000000096
PGSC0003DMB000000302
PGSC0003DMB000000591
PGSC0003DMB000000076
0 1 2 4 7 10 15 20 30 50 100 500
Number of AFLP markers per bin (upper boundaries)

Chromosome 8 (56.9 Mb)

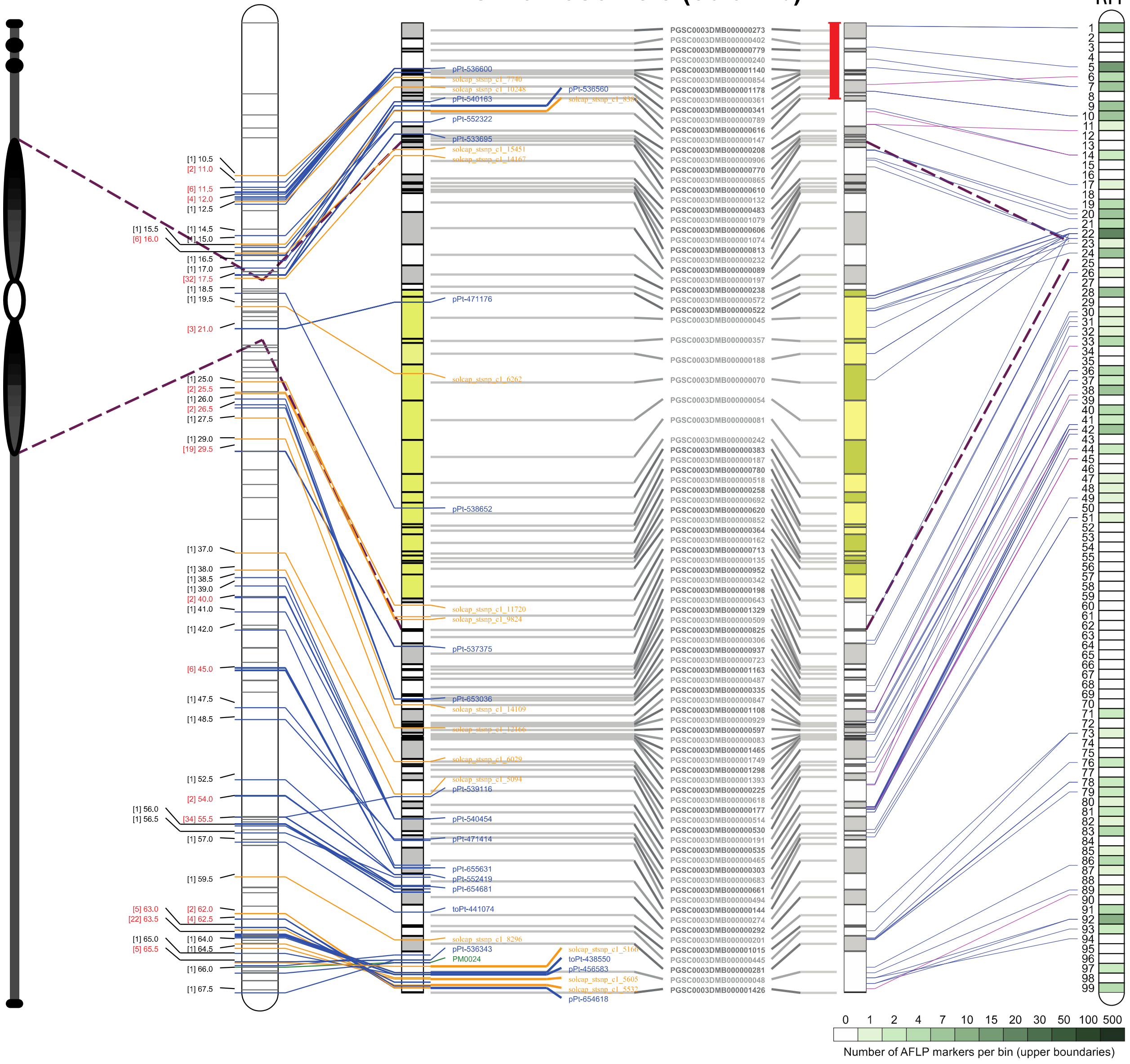

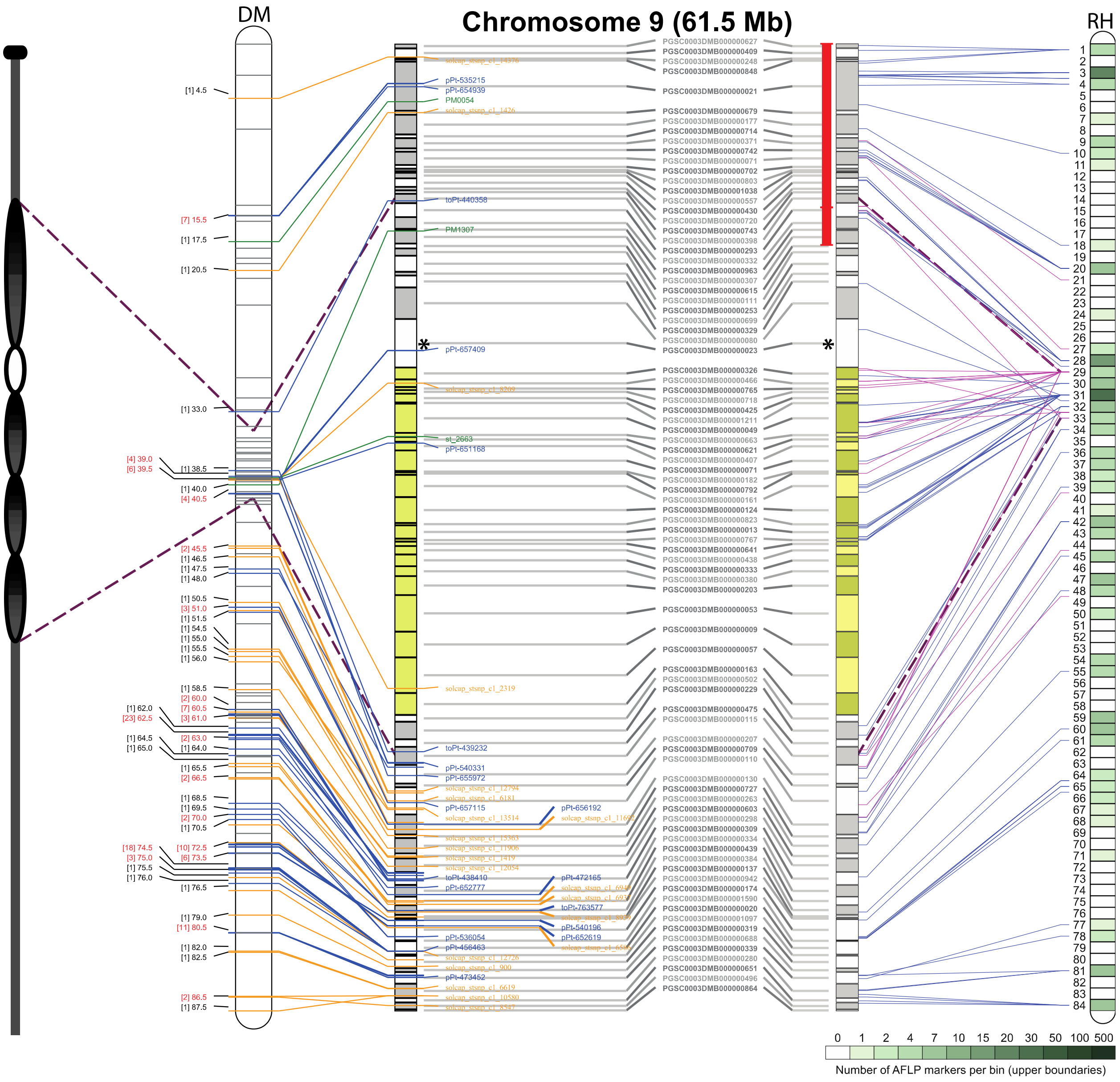

DM
Chromosome 9 (61.5 Mb)
RH
0 1 2 4 7 10 15 20 30 50 100 500
Number of AFLP markers per bin (upper boundaries)

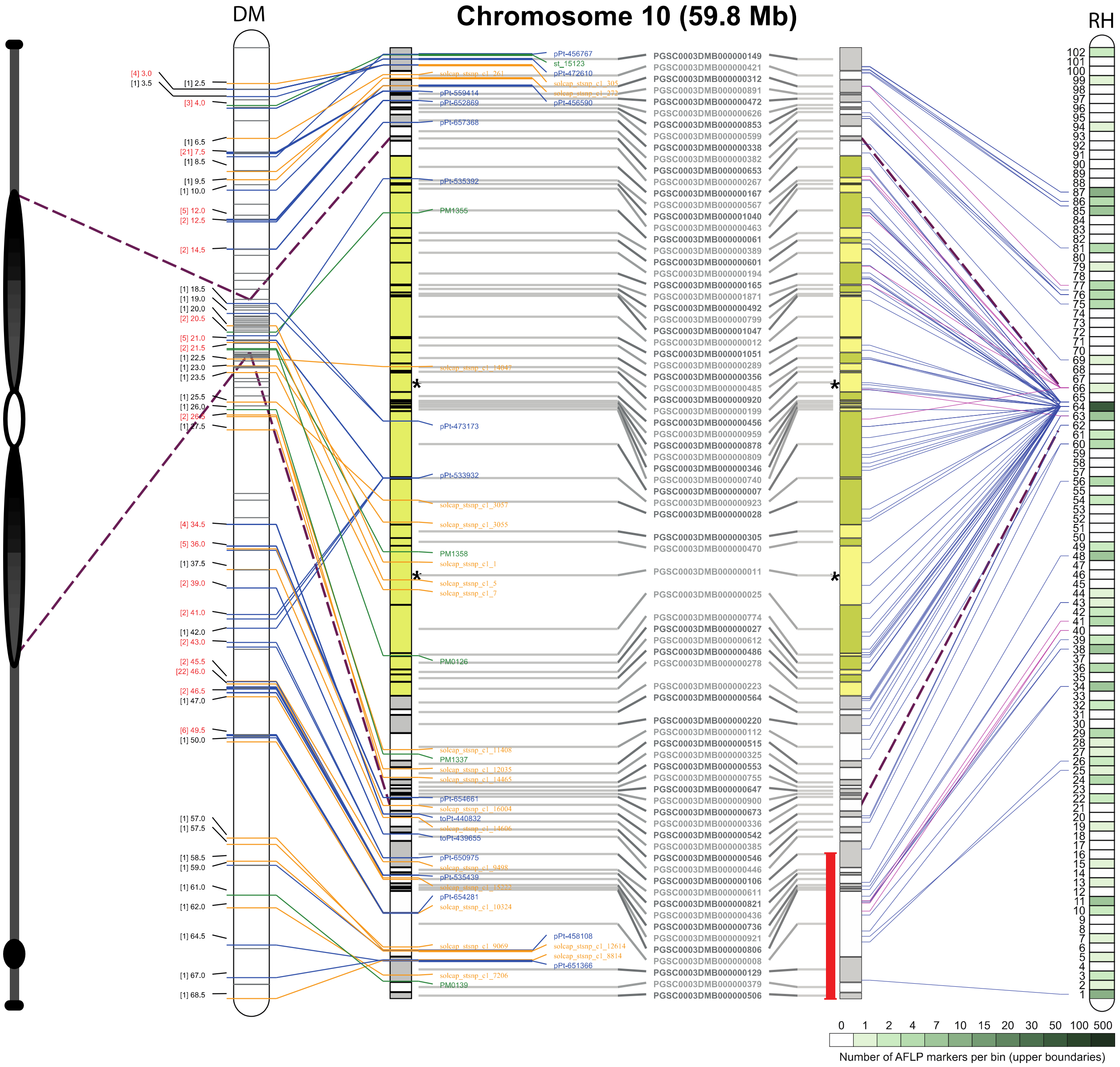
Chromosome 10 (59.8 Mb)
DM
RH
0 1 2 4 7 10 15 20 30 50 100 500
Number of AFLP markers per bin (upper boundaries)

Chromosome 11 (45.5 Mb)

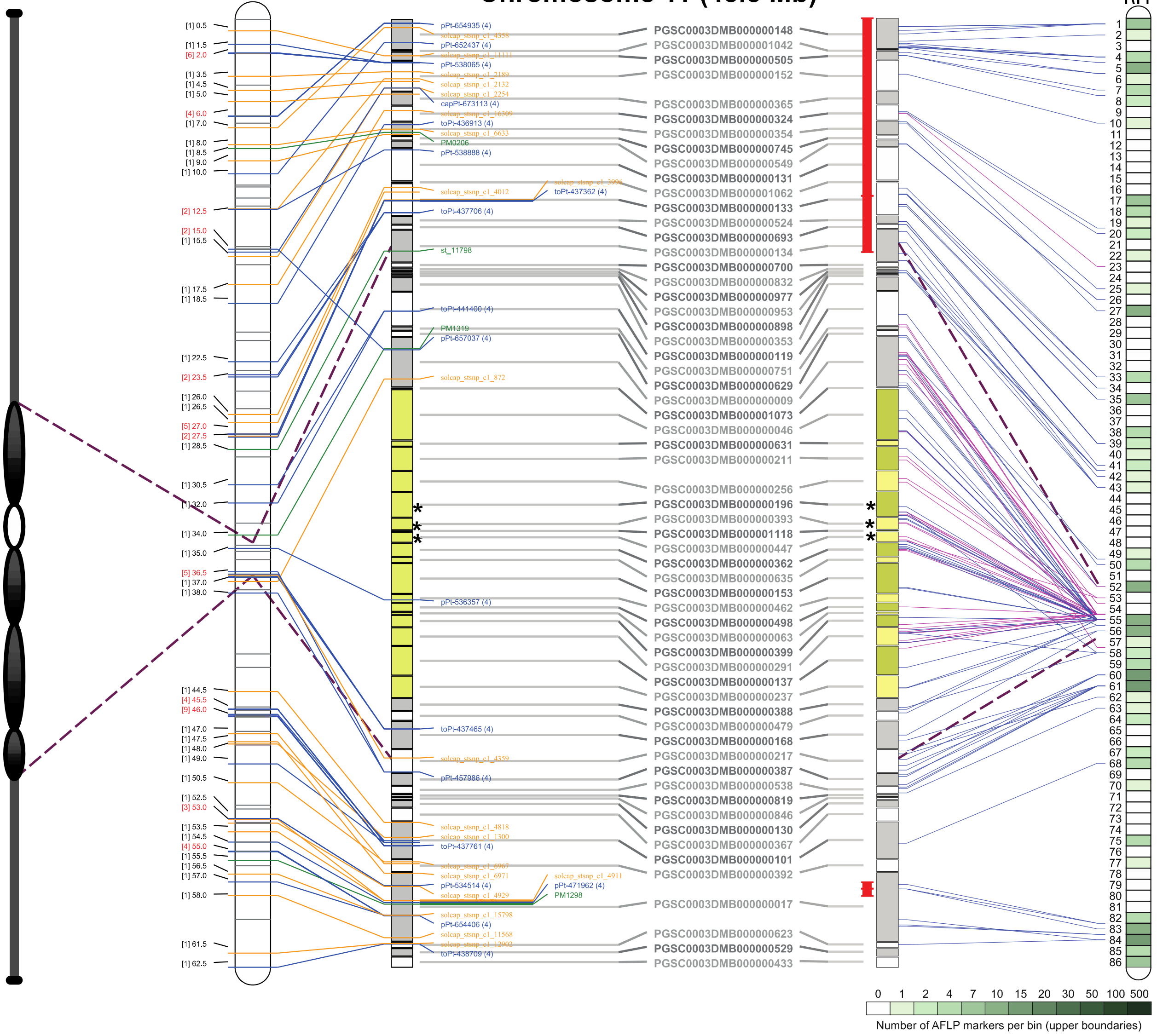

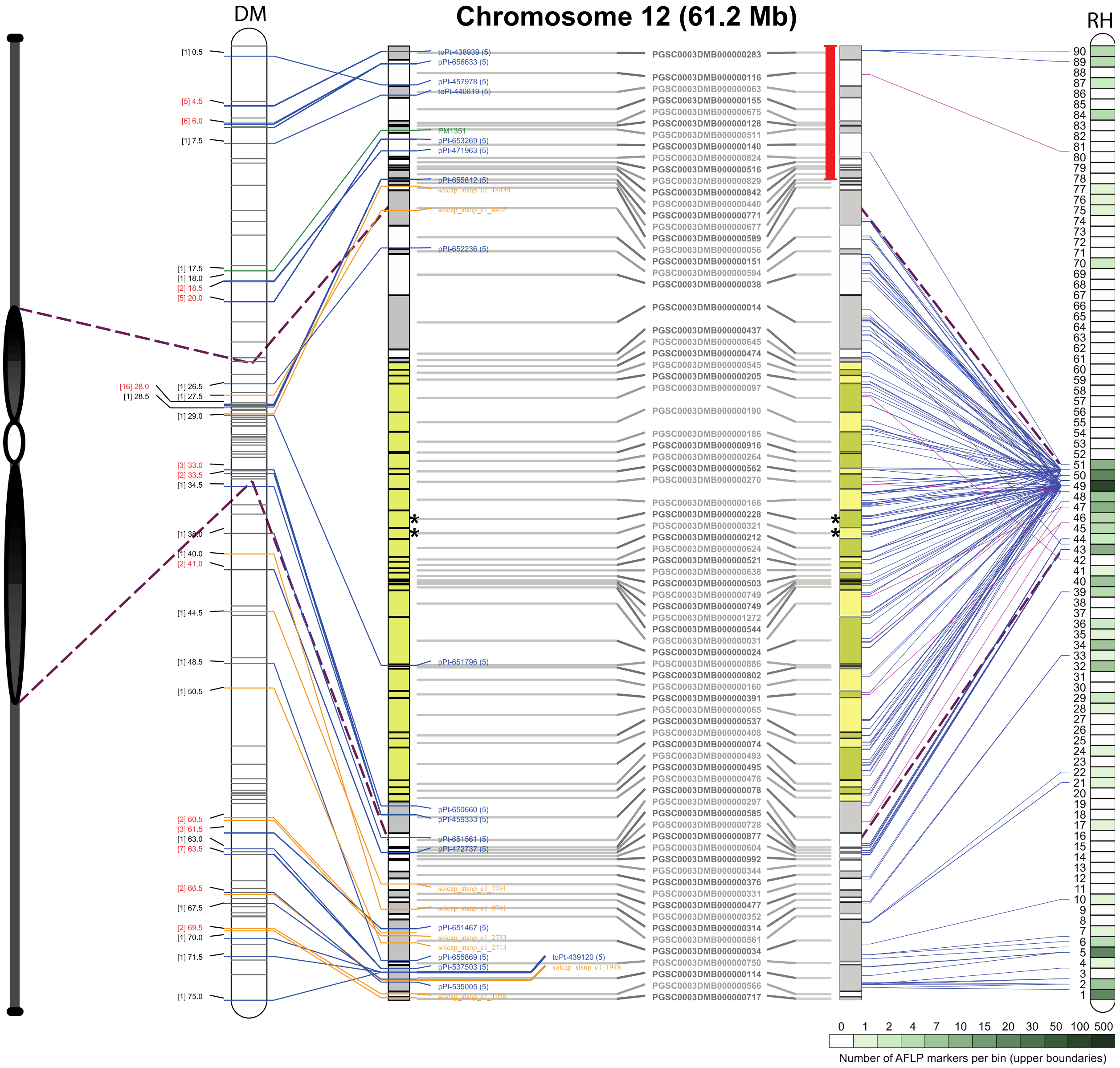

Figure S2 Illustration of the chromosome 2 - 12 pseudomolecules (PMs) integrated with the DM and RH genetic maps. STS and AFLP markers anchor sequence locations in the chromosome PMs to the DMDD and RH genetic maps, respectively. The AFLP marker positions in the PM were identified through sequence tag alignment of BAC clones from the RH WGP physical map. Superscaffolds comprising the PM are shown as alternating grey and white rectangular blocks. The layout of the PM for each of the genetic maps is shown separately but is identical with superscaffold IDs depicted in the middle. The pachytene idiogramis adapted from the potato reference genome publication (Potato Genome Sequencing Consortium 2011). The putative centromere region and pericentromeric/heterochromatic boundaries are demarcated by asterisks and dashed lines, respectively. Each DMDD marker type is colour coded: blue = DArTs, yellow = SNPs, green = SSRs. Blue and magenta lines emerging from the RH genetic map represent AFLP anchors and the intensity of green color corresponds to the AFLP marker density per bin as reported by Van Os *et al.* (2006). Magenta lines represent AFLP markers with a relatively inaccurate mapping position on the RH genetic map, covering an interval of 5 or more bins. Regions in the central heterochromatin where superscaffold order and orientation are not completely resolved are indicated in yellow. Inversions with the tomato sequence are indicated with red interval bars.
